# Replicate Me if You Can: Assessing Measurement Reliability of Individual Differences in Reading Across Measurement Occasions and Methods

**DOI:** 10.1111/cogs.70121

**Published:** 2025-12-30

**Authors:** Patrick Haller, Cui Ding, Maja Stegenwallner‐Schütz, David R. Reich, Iva Koncic, Silvia Makowski, Lena A. Jäger

**Affiliations:** ^1^ Department of Computational Linguistics University of Zurich; ^2^ Department of Computer Science University of Potsdam; ^3^ Department of Special Education University of Koblenz

**Keywords:** Individual differences, Naturalistic reading corpus, Eye‐tracking, Self‐paced reading, Two‐task Bayesian modeling, Measurement reliability, Sentence processing

## Abstract

Psycholinguistic theories traditionally assume similar cognitive mechanisms across different speakers. However, more recently, researchers have begun to recognize the need to consider individual differences when explaining human cognition. An increasing number of studies have investigated how individual differences influence human sentence processing. Implicitly, these studies assume that individual‐level effects can be replicated across experimental sessions and different assessment methods such as eye‐tracking and self‐paced reading. However, this assumption is challenged by the Reliability Paradox. Thus, a crucial first step for a principled investigation of individual differences in sentence processing is to establish their measurement reliability, that is, the correlation of individual‐level effects across multiple measurement occasions and methods. In this work, we present the first naturalistic eye movement corpus of reading data with four experimental sessions from each participant (two eye‐tracking sessions and two self‐paced reading sessions). We deploy a two‐task Bayesian hierarchical model to assess the measurement reliability of individual differences in a range of psycholinguistic phenomena that are well‐established at the population level, namely, effects of word length, lexical frequency, surprisal, dependency length, and number of to‐be‐integrated dependents. While our results indicate high reliability across measurement occasions for the word length effect, it is only moderate for higher‐level psycholinguistic predictors such as lexical frequency, dependency distance, and the number of to‐be‐integrated dependencies, and even low for surprisal. Moreover, even after accounting for spillover effects, we observe only low to moderate reliability at the individual level across methods (eye‐tracking and self‐paced reading) for most predictors, and poor reliability for predictors of syntactic integration. These findings underscore the importance of establishing measurement reliability before drawing inferences about individual differences in sentence processing.

## 1 Introduction

Theories of human sentence processing generally posit identical cognitive mechanisms involved in language processing for all native language users. However, within the history of cognitive science, the idea of idealized human cognition—understood as being identical across individuals—has not remained uncontested. Several researchers have emphasized that individual differences should play a more prominent role in theories of human cognition, given their potential impact on cognitive processes (e.g., Estes, 1956; Daneman & Carpenter, 1980; Levinson, 2012; Van Dyke, Johns, & Kukona, 2014). In particular, within the area of sentence processing, evidence has accumulated indicating that individual differences in cognitive capacities impact comprehension (Carter & Luke, 2018; Frinsel & Christiansen, 2024; Farmer, Fine, Misyak, & Christiansen, 2017; Nicenboim, Vasishth, Gattei, Sigman, & Kliegl, 2015; Staub, 2021; Vuong & Martin, 2014, inter alia). For example, Kuperman and Van Dyke (2011) showed that individual differences related to measures of cognitive control significantly impact how word length and lexical frequency affect readers' fixation times. Although longer and less frequent words typically lead to an increase of fixation durations, this effect is weaker for individuals with high cognitive control. Nicenboim et al. (2015) showed that in sentence regions with high memory load, readers with lower working memory capacity exhibit more regressive saccades—a measure that indicates greater processing difficulty—compared to readers with higher working memory capacity. Although these studies are typically cited to highlight the need to better understand how domain‐general cognitive capacities and language processing interact (Kidd, Donnelly, & Christiansen, 2018), we contend that they highlight the need to first determine whether individual differences in sentence processing can be reliably measured.

Naturally, most research on sentence processing has focused on establishing the population‐level validity of psycholinguistic theories, that is, on psycholinguistic phenomena that can be consistently observed at the population level. Such experimental studies typically measure psychological effects as differences in response to stimuli that systematically differ with respect to an experimental manipulation. Importantly, the between‐subjects variability of the measurement outcome for each condition must be low in order for the population‐level effect (i.e., the difference in measurements between conditions) to reach statistical significance. However, recent research on individual differences in sentence processing indicates that theories that hold at the population level might not generalize to every individual among the group of healthy adult native speakers. However, this finding rests on a common, yet untested assumption in studies investigating individual differences: the assumption that individual differences in sentence processing are stable over time. Claims such that speakers with low cognitive control exhibit stronger garden path effects than those with high cognitive control (Novick, Hussey, Teubner‐Rhodes, Harbison, & Bunting, 2014; Vuong and Martin, 2014; Woodard, Pozzan, & Trueswell, 2016) rely on the implicit assumption that individual differences are stable—that is, a speaker with high cognitive control will consistently show a below‐average garden path effect regardless of the specific time of testing. Along the same lines, it is often assumed that individual effects observed in an eye‐tracking (ET) experiment will correspond to those elicited in a self‐paced reading (SPR) experiment and vice versa. However, before integrating such findings into broader theoretical models, it is crucial to first test the reliability of these individual differences. Thus, establishing measurement reliability of individual‐level effects is a prerequisite for developing robust theories that account for individual variability in sentence processing. Only then can we go one step further and determine whether individual differences shape language processing qualitatively (through distinct mechanisms or strategies) or quantitatively (through modulation of shared mechanisms). Without this foundational step, theoretical interpretations may be premature or misleading.

In sum, from a methodological point of view, establishing the reliability of individual differences is a necessary first step toward their principled investigation (Parsons, Kruijt, & Fox, 2019). In other words, we need to ask whether individuals showing strong or weak sensitivity to a manipulation observed on one day (or with one method) exhibit similar above‐ or below‐average sensitivity when retested on another day (or with a different method). In this study, we operationalize reliability as the stability of individual‐level effects across measurements from multiple sessions with the same method or different methods. By testing reliability in this way, we aim to determine whether individual differences in sentence processing are stable over time and reproducible across distinct measurement occasions.

The assumption of stable individual differences across multiple measurement occasions is already called into question by the reliability paradox (Hedge, Powell, & Sumner, 2018). It states that the reliability of effects across multiple measurement occasions tends to be lower at the individual level for those effects that exhibit high between‐subjects reliability, which is necessary for strong replicability at the population level. Several studies have evaluated measurement reliability of individual differences in sentence processing using SPR or ET (Cunnings & Fujita, 2021; Carter and Luke, 2018; Frinsel & Christiansen, 2024; Henderson & Luke, 2014; Staub, 2021), providing empirical evidence for the paradox. However, most of these studies (except for Henderson & Luke, 2014 and Carter and Luke, 2018) have not tested individual differences in sentence processing across multiple measurement occasions, but rather correlated performance on two sets of stimuli that were presented within the same session. Moreover, to date, no study has comprehensively assessed measurement reliability across different methods, and existing studies on measurement reliability of individual differences in sentence processing are limited to English.

To offer a more comprehensive understanding of the measurement reliability of individual differences, we present Individual Differences Corpus (InDiCo), a large‐scale, multi‐session, and cross‐method naturalistic reading data set in German, combining data collected from two different laboratories. Our data set differs from previous research in several key ways: (i) it focuses on German, and hence constitutes the first study on the topic at hand that investigates a language other than English; (ii) it investigates naturalistic reading rather than minimal‐pair sentences in a planned experiment, and hence covers a larger range of linguistic constructions; (iii) contains reading data from the same participants across four experimental sessions including two ET and two SPR sessions; (iv) provides a comprehensive psychometric assessment of each participant; and (v) offers rich linguistic annotations for studying well‐established, key population‐level effects, such as word length, lexical frequency, predictability (surprisal), and dependency locality.

In our Bayesian analyses, we assess measurement reliability of individual‐level effects both across experimental sessions using the same method (measurement reliability across measurement occasions) and across methods (cross‐method reliability) by adapting Rouder and Haaf's (2019) hierarchical two‐task model. All data and code are available in an open data repository.^1^

### 1.1 Individual differences in sentence processing

The aim of psycholinguistic theories of sentence processing is to understand the cognitive mechanisms that underlie language comprehension and production. They are designed to explain empirically observed phenomena such as effects of predictability (including garden path effects), similarity‐based interference, (anti‐)locality effects, or illusions of grammaticality. However, it is crucial to note that these phenomena are observed at the population level, raising questions about their generalizability to each individual, such as: Does each individual exhibit such effects, or are there (groups of) readers who show deviating patterns, potentially reflecting different processing mechanisms or strategies? Already Estes (1956) pointed out that individual cognitive processing cannot be deduced from population‐level observations. Half a century later, Levinson (2012) issued a warning that neglecting individual differences in cognitive science could result in theories that promote a misleading notion of idealized human cognition that is largely invariant between individuals. Along the same lines, Kidd et al. (2018) expressed concerns that psycholinguistic theories and methods systematically downplay the role of individual differences. Indeed, it is a common practice in current psycholinguistic research to regard individual differences merely as a source of variance, controlled through hierarchical (e.g., linear‐mixed effects) statistical models, while primarily focusing on the population‐level (fixed) effects for theoretical interpretations of the results.

Although individual differences have been traditionally modeled as sources of noise in psycholinguistic research, recent studies highlight their importance to theories of sentence processing. A growing body of work has investigated how variability in participants' cognitive capacities interacts with population‐level psycholinguistic effects. For instance, individual differences in participants' cognitive capacities, such as working memory capacity or cognitive control, have been shown to interact with population‐level psycholinguistic effects in sentence processing. Most of these studies are correlational in nature, where scores obtained from psychometric tests are correlated with behavioral measures such as reading patterns. For example, individual differences in working memory capacity have been shown to play a role in the resolution of syntactic dependencies and affect well‐established phenomena such as locality and anti‐locality effects (Nicenboim et al., 2015), similarity‐based interference in reflexive processing (Cunnings & Felser, 2013), the preference for subject over object relative clauses (Farmer et al., 2017), preferences in the resolution of syntactic ambiguity (Just & Carpenter, 1992; Swets, Desmet, Hambrick, & Ferreira, 2007), or predictability effects (Haller, Bolliger, & Jäger, 2024; Ryskin, Levy, & Fedorenko, 2020). Moreover, working memory has been shown to affect general reading comprehension (Daneman & Carpenter, 1980; McVay & Kane, 2012) and interact with well‐established effects at the lexical level, such as effects of lexical frequency or word length (Kuperman & Van Dyke, 2011). Outside of sentence processing, working memory has also been shown to affect listeners' semantic‐pragmatic adaptation abilities (Schuster, Mayn, & Demberg, 2023). Cognitive control has been shown to affect sentence processing, for instance, reflected in garden path (Novick et al., 2014; Vuong and Martin, 2014; Woodard et al., 2016) and local attraction effects (Nozari, Trueswell, & Thompson‐Schill, 2016), but also word processing mechanisms such as lexical ambiguity resolution in children (Khanna & Boland, 2010) and interference between L1 and L2 in bilinguals (Festman, Rodriguez‐Fornells, & Münte, 2010).

Besides cognitive capacities, researchers have identified additional factors that affect language processing and reading, including, but not limited to, age (DeDe, Caplan, Kemtes, & Waters, 2004; Federmeier, Kutas, & Schul, 2010), fatigue (Siegenthaler, Bochud, Bergamin, & Wurtz, 2012), and exposure to specific linguistic structures such as structured practice with relative clauses in experimental settings (Wells, Christiansen, Race, Acheson, & MacDonald, 2009), or L2‐exposure (Tiffin‐Richards, 2024). Another study by Gordon, Moore, Choi, Hoedemaker, and Lowder (2020) showed that individual differences in reading experience correlate with specific variations in eye movement behavior during reading that are associated with the cognitive processes involved in word recognition.

Lastly, Kidd et al. (2018) pointed out that the extent to which human sentence processing is affected by individual differences, including environmental variables such as socioeconomic status, is probably *under*estimated since psycholinguistic experiments almost exclusively focus on a homogeneous subsample of the human population, namely, participants from WEIRD (western, educated, industrialized, rich, democratic; Henrich, Heine, & Norenzayan, 2010b, 2010a) societies.

From the body of work presented in this paragraph, we identify three types of studies that have contributed to our knowledge of individual differences in sentence processing: studies investigating static or context‐independent lexical variables such as word length and lemma frequency; studies on syntactic dependency formation; and studies on predictive processing where lexical and syntactic aspects interact. In the present study, our aim is to cover these three classes of phenomena and specifically examine the following five word‐level variables: word length, lemma frequency, syntactic integration of dependents (only for syntactic heads), dependency length (only for head‐initial dependents), and lexicalized surprisal. This selection allows us to examine a wide range of phenomena whose impact on reading times presumably originates from lower‐level oculomotor processes, as is the case for word length, to more context‐dependent, higher‐level processes such as dependency length.

### 1.2 The reliability paradox

As argued above, the measurement reliability of individual differences demands empirical support due to the reliability paradox described by Hedge et al. (2018). The reliability paradox highlights the difference between two concepts, both referred to as “reliability,” yet used differently in two subfields of psychology: Whereas in experimental research, an effect is referred to as reliable if it replicates across participants and studies (i.e., replicability at the *population level*), in correlational research, reliability refers to the consistency of a measure across occasions, typically quantified as the correlation between an individual's scores when the same test is administered more than once (i.e., reliability at the *individual level*). The paradox states that tests (or experimental manipulations) with high population‐level reliability often show lower reliability in terms of individual performance across measurement occasions.

**Fig. 1:**
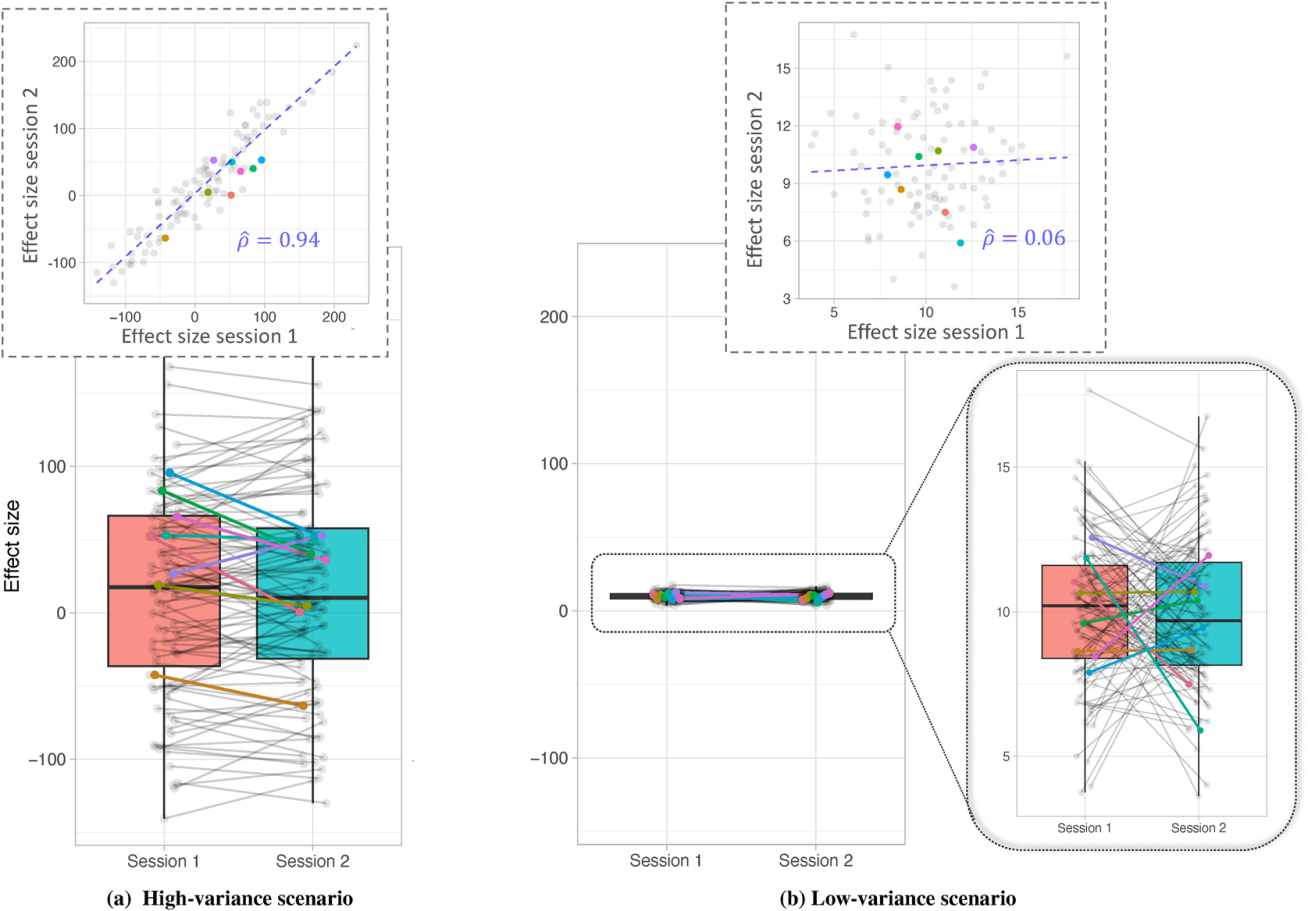
**Illustration of the Reliability Paradox (number of trials per subject = 5)**: Each pair of dots represents simulated effect sizes across two measurement occasions for a specific individual. For visualization, randomly selected participants are highlighted in color. As depicted in panel (a), high between‐subjects variance results in nonsignificant population‐level effects but high individual‐level reliability across measurement occasions, as indicated by a between‐sessions correlation of $\hat{\rho }=0.94$. By contrast, as depicted in panel (b), low between‐subjects variance results in statistically significant population‐level effects in both sessions with the estimated effect size being close to the true effect size of 10, but low individual‐level reliability across measurement occasions, as indicated by a between‐sessions correlation of $\hat{\rho }=0.06$. The reliability estimates are operationalized as Pearson correlation coefficients $\hat{\rho }$. The true correlation coefficient $\rho^{*}$ was set to 0.95.

We illustrate the relationship between population‐level variability and individual‐level variability using simulated data. Fig. 1 shows observed effect sizes x and y associated with some experimental manipulation of interest, obtained from the same participants at two separate measurement occasions. Each estimate is based on five trials for a sample of 100 participants. The true effect sizes $x^{*}$ and $y^{*}$ are assumed to have identical means, $\mu_{x^{*}}$ and $\mu_{y^{*}}$, and are sampled from a bivariate normal distribution, that is, $\left(\begin{array}{c} x^{*}\\ y^{*} \end{array}\right)∼N(\mu ,\Sigma )$, with mean $\mu =\left(\begin{array}{c} \mu_{x^{*}}\\ \mu_{y^{*}} \end{array}\right)$ and $\mu_{x^{*}}=\mu_{y^{*}}$, and variance‐covariance matrix $\Sigma =\left(\begin{array}{cc} \sigma_{x^{*}}^{2} & \sigma_{x^{*}y^{*}}\\ \sigma_{x^{*}y^{*}} & \sigma_{y^{*}}^{2} \end{array}\right)$, where $\sigma_{x^{*}}^{2}$ and $\sigma_{y^{*}}^{2}$ represent the between‐subjects variance of $x^{*}$ and $y^{*}$, respectively, and $\sigma_{x^{*}y^{*}}$ denotes their covariance. We set the mean to μ=10, and the variance covariance matrix to $\sigma_{x^{*}}^{2}=\sigma_{y^{*}}^{2}=80$ in the high‐variance scenario and to 1 in the low‐variance scenario. To simulate two measurement occasions (i.e., experimental sessions), we add independent measurement error, sampled from N(0,5), to $x^{*}$ and $y^{*}$, yielding the observed measurements x (observed effect size in session 1) and y (observed effect size in session 2).^2^

As illustrated in Fig. 1, the high‐variance scenario (left panel) results in an uncertain population‐level effect (it might even be positive *or* negative), but shows high reliability in terms of the individual‐level effects across the two sessions ($\hat{\rho }=0.94$, see derivation below). Conversely, in the low‐variance scenario on the right‐hand side, the effect sizes in both sessions show robust effect estimates across participants around 10. However, at the same time, the reliability of the individual effects across the two sessions is low ($\hat{\rho }=0.06$). These contrasting outcomes raise the question: why does high between‐subjects variance yield high individual‐level reliability, while low between‐subjects variance yields low individual‐level reliability? To address this question, we examine the relationship formally.

**Attenuation of correlations under measurement error**

The **true** Pearson correlation coefficient $\rho^{*}$ between $x^{*}$ and $y^{*}$ is given by

(1) $$\rho^{*}=\frac{\sigma_{x^{*}y^{*}}}{\sqrt{\sigma_{x^{*}}^{2}\sigma_{y^{*}}^{2}}}$$

and quantifies the degree to which the individual effects replicate over the two sessions. Regardless of the true effect sizes $x^{*}$ and $y^{*}$ and the experimental technique used for the measurement, the measurement process always includes some noise or uncertainty. This random error is, unlike systematic errors, inherently nonreproducible, but it can be statistically modeled. Therefore, the observed variables, denoted as x and y, are distinct from their error‐free true counterparts $x^{*}$ and $y^{*}$, which cannot be observed directly. Formally, x and y are composed of the true $x^{*}$ and $y^{*}$ plus some measurement error, such that x= $x^{*}$ + $\varepsilon_{x}$ and y= $y^{*}$ + $\varepsilon_{y}$, where $\varepsilon_{x}∼N\left(0,\sigma_{\varepsilon_{x}}^{2}\right)$ and $\varepsilon_{y}∼N\left(0,\sigma_{\varepsilon_{y}}^{2}\right)$. Note that in this additive error model, $\varepsilon_{x}$ and $\varepsilon_{y}$ are independent of each other and also independent of $x^{*}$ and $y^{*}$.

As a consequence, the true correlation coefficient $\rho^{*}$ can only be approximated by estimation from the observed experimental data. We can attempt to approximate the true correlation coefficient $\rho^{*}$ with a sample correlation ${\hat{\rho }}_{N}$, calculated as:

(2) $${\hat{\rho }}_{N}=\frac{\sum_{i=1}^{N}\left(x_{i}-\bar{x}\right)\left(y_{i}-\bar{y}\right)}{N\sqrt{s_{x}^{2}\;s_{y}^{2}}},$$

where i∈{1,⋯,N} denotes one out of N participants. $x_{i}$ and $y_{i}$ refer to the observed effect in a first and a second experimental session, respectively, for a given participant i; $\bar{x}$ and $\bar{y}$ refer to the sample means, and $s_{x}^{2},s_{y}^{2}$ to the sample variances. ${\hat{\rho }}_{N}$ approximates the population value

(3) $$\rho =\frac{Cov(x,y)}{\sqrt{Var(x)}\sqrt{Var(y)}},$$

that is, it holds that $\lim_{N\to \infty }{\hat{\rho }}_{N}=\rho$. While intuitively, one might think that the measurement error would vanish such that $\rho =\rho^{*}$, in reality, under the above introduced additive error model, ρ is biased downward, that is, $\rho <\rho^{*}$ for any measurement error $\sigma_{\varepsilon_{x}}^{2}$, $\sigma_{\varepsilon_{y}}^{2}>0$. Formally, this is shown by the following relationship, derived already by Spearman (1904):

(4) $$\rho =\frac{1}{\sqrt{\left(1+\frac{\sigma_{\varepsilon_{x}}^{2}}{\sigma_{x^{*}}^{2}}\right)\left(1+\frac{\sigma_{\varepsilon_{y}}^{2}}{\sigma_{y^{*}}^{2}}\right)}}\rho^{*}.$$

Thus, the larger the ratio of the measurement error $\sigma_{\varepsilon_{x}}^{2}$ to the true between‐subjects variance $\sigma_{x^{*}}^{2}$, the larger this bias. This explains the low individual‐level reliability (i.e., between‐sessions correlation) in the low‐variance scenario: the ratio of the error to the true between‐subjects variance is 70 times higher. This measurement error significantly obscures the differences in the magnitudes of the effects within the group in a given session. As a result, these magnitudes no longer correspond across sessions, and the true variance between participants is overshadowed.

**Increasing the number of trials**

In the above simulation, individual effect sizes were estimated based on five trials only. Although the influence of trial count on study outcomes depends on the specific effect under investigation (Hedge et al., 2018; Staub, 2021), Rouder et al. (2023) emphasized its critical role. They argue that too few trials can introduce systematic biases, while too few participants contribute to unsystematic noise. Increasing the number of trials per task, therefore, can enhance the reliability of individual‐level effects, thus effectively mitigating the limitations posed by the reliability paradox.

**Fig. 2:**
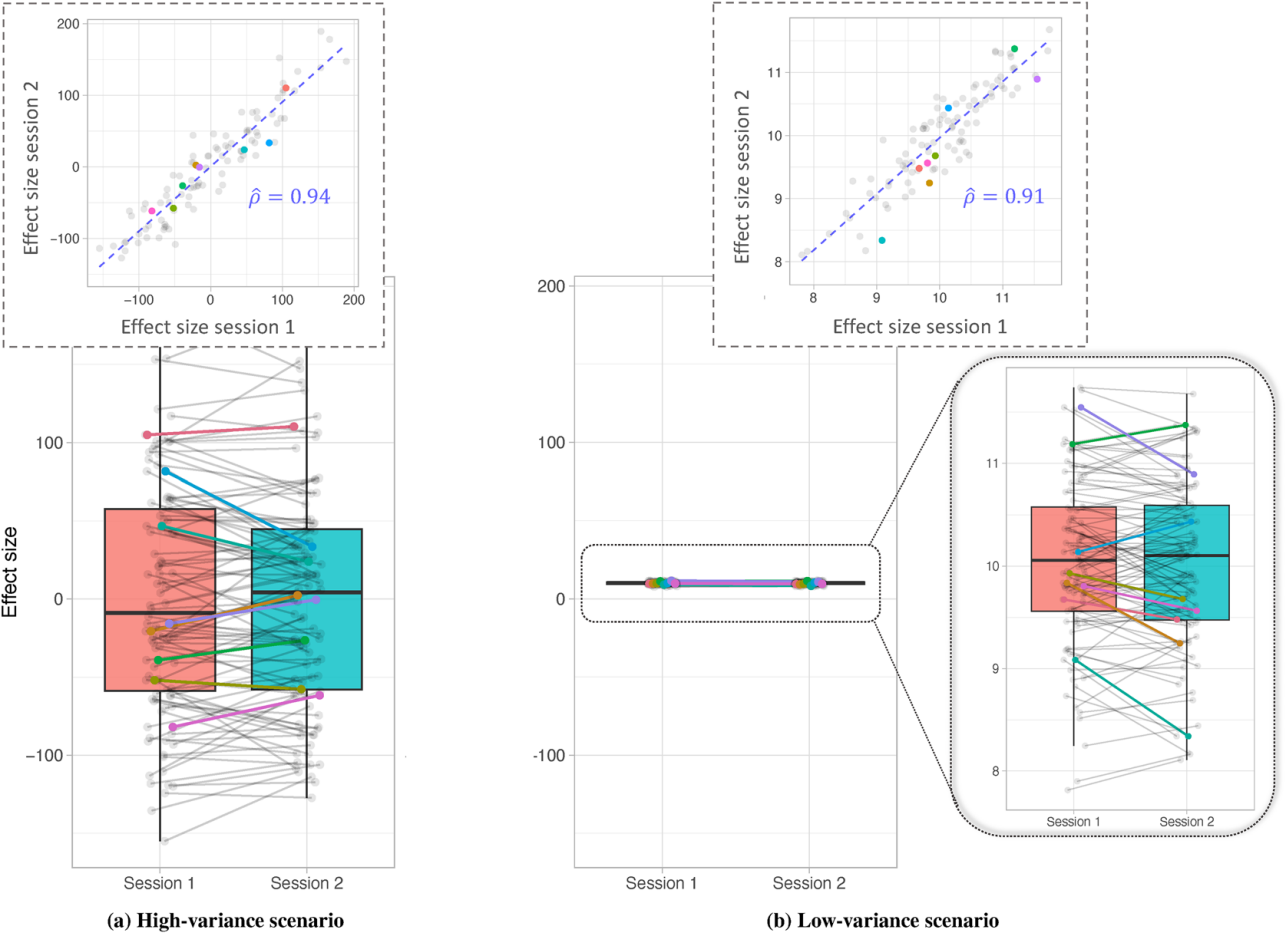
**Overcoming the Reliability Paradox (number of trials per subject = 3000)**: Each pair of dots represents simulated effect sizes across two measurement occasions (sessions) for a specific individual who reads 3000 words in each session. For visualization, randomly selected participants are highlighted in color. In contrast to Fig. 1, the low between‐subjects variance depicted in panel (b) results not only in significant population‐level effects but also high individual‐level reliability of the effect over sessions. The reliability estimates are operationalized as correlation coefficients $\hat{\rho }$. The true correlation coefficient $\rho^{*}$ was set to 0.95.

To illustrate this, we re‐ran our simulation increasing the number of trials completed by each participant to 3000 while keeping all other settings unchanged. The results presented in Fig. 2 show that, although the high‐variance scenario depicted in panel (a) remains largely unchanged, the individual effect sizes in the low‐variance scenario depicted in panel (b) are not only more tightly clustered, yielding statistically significant effects at the population level (p<2e−16), but also exhibit reliable individual‐level effects ($\hat{\rho }=0.91$). We provide a formal proof for why this relationship holds in Online appendix A.

With regard to the number of trials, naturalistic reading offers an advantage over minimal‐pair experiments precisely because each word can be conceptualized as a single trial (Rouder et al., 2023). Thus, in a naturalistic reading scenario, participants easily contribute thousands of trials, which makes the choice of 3000 trials in the simulation above a realistic approximation. In minimal‐pair experiments, the measure of interest is often represented by the reading time of a limited area of interest within a sentence, therefore, the number of trials equals the number of items per experiment. This makes naturalistic reading a suitable and worthwhile approach for studying individual differences in sentence processing for effects that can be measured at every word.^3^

### 1.3 Disentangling measurement reliability in reading experiments

The reliability of reading measures, or any other measure, reflects the degree to which results are consistent across different occasions of testing or across different methods. This can be operationalized as the by‐subject correlation between two arrays of measurements, $x^{u}$ and $x^{v}$, recorded at measurement occasions (or using measurement methods) u and v. Generally speaking, reliability depends on (i) how the measures $x^{u}$ and $x^{v}$ relate to each other (e.g., whether in both experimental sessions, the same test or method that generated the measurements x was used) and (ii) the temporal relationship between u and v. In the following, we will first introduce the concepts of measurement reliability across measurement occasions and measurement reliability across methods. Then, we will examine the reading measures used to quantify reliability, each of which reflects a different aspect of the reading process, as well as the measures' relationship to cognitive processes. Finally, we will review the modeling approaches based on which reliability is assessed.

#### 1.3.1 Measurement reliability of individual differences across measurement occasions

*Test‐retest reliability* captures the consistency of measurements over time, that is, u and v are temporally spaced experimental sessions. Technically, the corresponding reliability coefficient is computed by correlating repeated measures, that is, responses to the exact same stimuli collected at different occasions of testing. However, in reading comprehension tasks, re‐reading texts poses challenges due to memory effects that can alter eye movements and comprehension accuracy (Meiri & Berzak, 2024). Previous studies have addressed this issue using *split‐half reliability*. This reliability measure is computed by correlating measurements from two halves of an item set, both obtained during the same measurement occasion (James, Fraundorf, Lee, & Watson, 2018; Staub, 2021), that is, u and v are the same experimental session. While this approach captures how reliably individual differences are measured across items, it does not assess their stability across measurement occasions, which is the central focus of our study. For this reason, we apply *parallel‐forms reliability*, assessing the stability of an individual's reading behavior across *separate measurement occasions*.^4^ Technically, we correlate each individual's observed reading measures on texts presented in an experimental session u with measures on different texts presented in a second, temporally spaced experimental session v, ensuring that the texts measure the same underlying construct.^5^ Importantly, using parallel‐forms reliability allows us to estimate reliability while counterbalancing the order of texts. This is essential to minimize potential ordering biases. In summary, this approach ensures accurate reliability estimates while also maintaining a counterbalanced design.

#### 1.3.2 Cross‐method reliability of individual differences

ET and SPR are the most commonly used experimental methods in psycholinguistic research on sentence processing (Just, Carpenter, & Woolley, 1982). SPR times or reading measures extracted from ET data are known to reflect processing ease experienced at the currently read word or phrase. Both methods are used with the same type of experimental materials and task, that is, a stimulus sentence or text (e.g., Futrell et al., 2021) followed by a comprehension question, and are often treated as interchangeable. Therefore, the selection of the method often only depends on the available lab infrastructure rather than the research question, and literature reviews or meta‐analyses have even pooled the data obtained from these two methods (Jäger, Engelmann, & Vasishth, 2017). Although SPR experiments are inexpensive and straightforward to implement, the resulting reading behavior is less naturalistic and provides only a single measure per word (or area of interest). In contrast, ET data allow the extraction of a wide range of reading measures, such as first‐fixation duration (a first‐pass measure) or re‐reading time (a late measure), some of which have been shown to align with SPR times (Boyce, Futrell, & Levy, 2020; Frank, Monsalve, Thompson, & Vigliocco, 2013; Witzel, Witzel, & Forster, 2012). However, notable differences emerge regarding the timing of these measures and the sensitivity to psycholinguistic manipulations, such as attachment preferences or the resolution of coordination ambiguities (Witzel et al., 2012). For example, ET tends to capture more immediate effects at disambiguation points, while SPR often shows spillover effects on subsequent words. Crucially, to the best of our knowledge, the degree to which participants adopt different reading strategies or exhibit individual differences across experimental methods has not been investigated yet. In other words, it is still unknown whether a participant who shows a strong sensitivity (i.e., an above‐average effect size) in response to a certain psycholinguistic manipulation with ET will show a similar sensitivity when tested with SPR. Given the above‐mentioned differences between ET and SPR, in addition to establishing the reliability of individual differences across measurement occasions, it is necessary to demonstrate cross‐method reliability of individual differences in sentence processing, especially if we aim to build psycholinguistic theories that account for principled, potentially qualitative, differences between individuals. Such theories require that the respective psycholinguistic manipulations show consistent effects across methods before any meaningful generalizations about individual variation can be drawn.

#### 1.3.3 Selecting reading measures

One crucial factor to keep in mind when examining cross‐method reliability is the relationship between the different measures obtained from both ET and SPR and the cognitive processes they reflect. As already mentioned in Section 1.3.2, there exist a wide range of reading measures that aggregate fixations occurring at different stages of the reading process. On the word‐level, we can distinguish between *first‐pass*, *later‐pass*, and *global* measures (Mézière, Yu, Reichle, von der Malsburg, & McArthur, 2023; Rayner, 2009), which have been tied to different *processes* in sentence processing.^6^

First‐pass measures such as first‐pass reading time or first‐fixation duration capture rapid processes, including word recognition and lexical access. These processes can be influenced by various properties of words, including their length, frequency, and predictability from context (Clifton et al., 2007).

On the other hand, effects observed during ambiguity resolution (Van Gompel & Pickering, 2001), syntactic misanalysis (Clifton et al., 2007), or at implausible continuations (Rayner, Warren, Juhasz, & Liversedge, 2004) often appear in later‐pass measures such as re‐reading times or regression counts due to readers re‐visiting previous material in an attempt to reconcile their initial interpretations with the continuation of the sentence or text (Pérez, Joseph, Bajo, & Nation, 2016).

Certain reading measures aggregate processing across multiple passes and are, therefore, considered global measures. For instance, total fixation time (TFT) sums the durations of all fixations on a word, including both initial and later‐pass fixations, thus capturing both early lexical processing and later integrative or corrective processes. Similarly, regression‐path duration (RPD), also known as go‐past time, includes all fixations from the first encounter with a word until the eyes move past it to the right, incorporating all regressions to earlier parts of the text. As such, global measures like TFT and RPD reflect the cumulative processing cost associated with a word and its integration into the sentence context. Importantly, while first‐pass measures cannot reflect late‐stage processes, it is still possible for later‐pass measures to reflect early‐stage processes due to potential delays in the oculomotor response.

Compared to ET, which provides multiple distinct measures reflecting the time course in which words are fixated, SPR only yields a single reaction time per word or region. This puts a limit on the temporal resolution at which cognitive processes during sentence processing can be inferred from SPR data. Moreover, the types of cognitive process reflected in the recorded reaction times depend on the specific paradigm. In our study, we used the cumulative window SPR paradigm, in which each word remains on the screen after its initial presentation, allowing participants to re‐inspect previously read text. However, since regressions are not recorded, this variant precludes distinguishing between cognitive processes reflected in first‐pass, later‐pass, and global measures. Another commonly used SPR variant is the moving‐window paradigm, where each subsequent word is revealed while masking the previous one. In this setup, regressions to earlier words are not possible. Recently, a more naturalistic extension of SPR, “bidirectional self‐paced reading” (BSPR), has been proposed (Paape & Vasishth, 2022), which allows participants to regress to earlier words or regions. A final limitation of SPR is its susceptibility to spillover effects, where processing difficulties on one word lead to slowdowns in reaction times on subsequent words (Boyce et al., 2020; Smith & Levy, 2013; Witzel et al., 2012), complicating the precise localization of processing difficulty. Overall, regardless of the paradigm, the constraints on natural reading behavior and temporal dynamics make SPR measures substantially different from ET measures.

#### 1.3.4 Modeling approaches to estimate measurement reliability

Having considered different types of measurement reliability and reviewed different reading measures, we now turn to the question of how reliability can be estimated in practice. Traditionally, the way to establish reliability for a given effect is to directly compute correlation coefficients of individual effect estimates across the two measurement occasions (or methods). This approach suffers from several short‐comings: First, it requires the same number of trials in the two sessions, and second, it conflates two independent sources of variability—between‐subjects variability on the one hand, and trial‐by‐trial variability on the other hand. In our simulation (see Section 1.2), between‐subjects variability was operationalized as the between‐subjects variance $\sigma_{x^{*}}^{2}$, $\sigma_{y^{*}}^{2}$, and trial‐by‐trial variability as the measurement error variance $\sigma_{\varepsilon_{x}}^{2}$, $\sigma_{\varepsilon_{y}}^{2}$. As illustrated by Eq. 4, the ratio between these two terms determines the accuracy of the reliability estimates. Using a hierarchical modeling approach allows us to disentangle these two sources of variability which is not possible with conventional approaches (Rouder & Haaf, 2019). Another advantage of hierarchical models is that they offer the flexibility of incorporating additional sources of variability, such as differences in baseline reading times across methods, or including spillover effects. For these reasons, in the present work, we use a hierarchical modeling approach to estimate reliability as detailed in Section 3.2.

### 1.4 Research questions

In this study, we investigate the measurement reliability of a range of lexical and sentence‐level psycholinguistic effects that are well‐established at the population level and constitute the foundation of many psycholinguistic theories. To this end, we collect a multi‐session, cross‐method naturalistic reading corpus in German with comprehensive psychometric assessments of participants, including measure of working memory capacity, cognitive control, intelligence, and reading fluency. This selection of psychological constructs was motivated by the body of work presented in Section 1.1, as these variables have been shown to impact sentence processing in earlier studies. We then assess the measurement reliability of individual differences in lexical‐ and sentence‐level psycholinguistic phenomena of interest (word length, lexical frequency, surprisal, dependency distance, number of left dependents) across multiple experimental sessions of the same method (i.e., *measurement reliability* of individual differences *across measurement occasions*) and *across methods* by comparing individual‐level effect estimates obtained from SPR and ET (i.e., *cross‐method reliability* of individual differences). We further contextualize our model results by assessing the relative contributions of trial‐by‐trial variability and between‐subjects variability of the individual effects.

## 2 The Individual Differences Corpus

The InDiCo is a multi‐session German naturalistic reading corpus and was collected in two different laboratories at the University of Zurich, Switzerland and the University of Potsdam, Germany, respectively. While the reading experiments in both laboratories were conducted under identical conditions (same technical setup, texts, and comprehension questions), they differ in terms of methods that were used (only ET in Potsdam vs. ET and SPR in Zurich; see Table 1).

- The Zurich reading experiment was conducted across four sessions: two ET sessions and two SPR sessions. The data are complemented with individual psychometric scores obtained from a wide range of psychometric assessments (see Section 2.3.2).
- In the Potsdam reading experiment, ET was used in all four sessions. Two sessions were conducted under regular conditions, two under adverse conditions.^7^ For the present study, we only use the reading data from the two regular sessions, that is, no data from the sessions under adverse conditions.

**Table 1:** InDiCo data: We collected data across two different testing sites (Zurich and Potsdam) using the same technical setup

Test site	ET sessions	SPR sessions	Psychometric assessment	Participants
Zurich	2	2	✓	65 (SPR), 63 (ET)
Potsdam	2	0	✗	68

*Note*. In Zurich, we collected data from each participant in four separate sessions (two eye‐tracking sessions and two self‐paced reading sessions). With each participant, we additionally conducted a comprehensive battery of psychometric tests. In Potsdam, we only collected eye‐tracking data (no self‐paced reading data), and no psychometric assessment was conducted. For five participants tested in Zurich, no eye‐tracking data is available due to calibration issues.

We also provide extensive lexical and syntactic annotations of the stimulus materials (see Section 2.2.2). In summary, our corpus allows for the study of individual differences in reading, be it for intra‐ or cross‐method comparisons, be it for analyses within participants or across participants.

### 2.1 Participants

A total of 136 native German speakers (83 female, 52 male, 1 nonbinary), aged 17–63 years (mean = 25.9, sd = 8.2), with normal or corrected‐to‐normal vision, participated in the experiments conducted in Zurich and Potsdam. In total, InDiCo includes 392 experimental sessions, 262 of which are ET and 130 are SPR sessions.

**Zurich**

Sixty‐eight participants were recruited via an online platform from the University of Zurich targeted at students as well as the local population in the Zurich area. They completed four experimental reading sessions (two ET, two SPR) along with the psychometric assessment distributed across the four sessions, with a time lag of about 1 week between any two sessions. Participants were reimbursed with CHF 120. Out of the 68 participants, 60 completed all four sessions.

**Potsdam**

Sixty‐eight participants were recruited via advertisements and online platforms, targeted at students as well as the local population in the Potsdam area. They participated in four experimental sessions (all ET), scheduled roughly 1 week apart. Unlike in Zurich, two out of four sessions were conducted under adverse conditions (see footnote 7), which is why the Potsdam ET data only consist of two ET sessions. They were reimbursed 50 EUR for all four sessions.

### 2.2 Reading materials

#### 2.2.1 Selection and presentation of the materials

A total of 16 texts taken from former TestDAF German proficiency assessments (TestDaF‐Institut, 2002) were used. Each text was split across six screens; one screen for the header and the remaining five for the text. Upon switching to the next screen, participants were not able to revisit previously displayed screens. Each text was followed by the 10 original TestDaF comprehension questions. Half of the texts were followed by multiple‐choice questions with three options, the other half with *yes*/*no*/*text doesn't say* questions. Unlike the original paper‐based TestDaF, participants could not refer back to the text when answering comprehension questions, and each question was presented on a separate screen. This presentation mode was chosen to ensure comparability of our data with typical psycholinguistic data sets where eye movements during reading reflect general reading processes, independent of specific comprehension questions and the associated answer‐seeking eye movement behavior. Along the same lines, not displaying the text together with each comprehension question yields the psycholinguistically desired comprehension scores that reflect text comprehension during the initial reading rather than the ability to search for answers during post hoc re‐reading.

#### 2.2.2 Linguistic annotation of the materials

All stimulus texts were annotated with the following word‐level linguistic features: word length (number of characters), log lexical (lemma) frequency, surprisal, part‐of‐speech (PoS) tags, and syntactic dependency relations. From the dependency relations, we calculated two features: (i) dependency distance, which is the distance of a syntactic dependent to its head,^8^ and (ii) number of left dependents of each syntactic head, or, in other words, the number of dependencies that need to be completed at the current word (besides the dependency to the head of the current word, which is captured by the first feature).

Lemma frequencies were extracted from dLexDB.^9^ Surprisal values were derived using a pretrained GPT2 model from HuggingFace.^10^ Since GPT‐2 employs tokenizers that split white‐space separated words into sub‐word tokens (Sennrich, Haddow, & Birch, 2016), we computed word‐level surprisal by summing up the surprisal values of the sub‐word tokens, which is a common practice (Giulianelli et al., 2024; Wilcox, Gauthier, Hu, Qian, & Levy, 2020, inter alia). PoS‐tags and dependency relations were automatically extracted with SpaCy (Honnibal, Montani, Van Landeghem, & Boyd, 2020) using the German transformer pipeline.^11^ Dependency relations are based on the TIGER treebank (Dipper, Brants, Lezius, Plaehn, & Smith, 2001) and use PoS‐tags according to the Stuttgart‐Tübingen tagset (STTS, Schiller, Teufel, & Thielen, 1999), and were subsequently corrected manually by a linguistically trained expert.

### 2.3 Experiments and procedure

#### 2.3.1 Reading experiment

Each participant read 16 texts, divided into four sets, and counterbalanced across the four sessions, such that each unique permutation of texts across sessions and the chronological order of their presentation within a session was assigned to only one participant. Within each session, half of the texts were followed by multiple‐choice questions and the other half by yes/no questions. Participants began the reading experiment by pressing the space bar. Upon advancing to the next screen—displaying the next portion of text—they were instructed to first focus on a fixation point aligned with the position of the first letter. They then read the text at their own pace, advancing through subsequent screens with the space bar, without the option to return to previous screens. On average, each screen contained 112(±18) words, each text contained 559(±30) words, each session contained 2233(±62) words. Drift check and, if necessary, drift correction was performed before the first screen of each text. After each text (six screens, including the title screen), participants answered 10 comprehension questions. At the end of a trial (i.e., after the comprehension questions and before the next text), they were allowed to take a break and re‐calibration was conducted if necessary.

In the SPR sessions, participants advanced through each text one word at a time by pressing the space bar. Previously displayed words remained on the screen until all words on a given screen had been presented (cumulative window paradigm).

For both methods, stimuli were presented in black Courier New mono‐spaced font (13×27 px per character) on a light gray background with double line spacing. Each character covered approximately $0.28^{\circ }$ of visual angle, corresponding to about 3.6 characters per degree.

#### 2.3.2 Psychometric assessment

In Zurich, each participant completed a comprehensive psychometric assessment of individual cognitive capacities. It included the assessment of verbal and nonverbal cognitive control, verbal and nonverbal working memory capacity, verbal and nonverbal intelligence (incl. vocabulary size), as well as lexical and nonlexical reading fluency. Table 2 provides an overview of all psychometric tests. Detailed test descriptions can be found in Online appendix C.

**Table 2:** Psychometric assessment: Tests were conducted with 65 participants recruited from Zurich

Cognitive capacity		Test/Task	DV(s)	Score type
*Cognitive Control*	V	Stroop	RT; acc	R
	NV	Simon	RT; acc	R
	NV	FAIR‐2	K‐value	P
*Working Memory*	V	Sentence span	acc	R
	NV	Operation span	acc	R
	NV	Memory updating	acc	R
	NV	Spatial short‐term memory	acc	R
*Intelligence*	V	MWT‐B	acc	P
	V	RIAS 1: Question answering	acc	P
	V	RIAS 3: Sentence completion	acc	P
	NV	RIAS 2: Excluding mismatching images,	acc	P
		finding missing elements in an image		
	NV	RIAS 4: Visual search of missing elements	acc	P
*Reading Fluency*	L	SLRT‐II, word reading	acc	P
	NL	SLRT‐II, pseudo‐word reading	acc	P

*Note*. *DV(s)* refers to dependent variables; *RT* refers to response latency, acc refers to response accuracy, V refers to verbal, NV refers to nonverbal, L refers to lexical, and NL refers to nonlexical. K‐value denotes the attention continuity measure defined in Section C.1.2. Score type indicates whether the final score represents a raw score (R), or percentiles (P) obtained from a norm table. Please see Appendix C for detailed test descriptions.

Throughout the reading experiments, we repeatedly assessed participants' wakefulness levels using the Karolinska Sleepiness Scale (Baulk, Reyner, & Horne, 2001), on which participants indicate the level that best reflects their current psychophysical state, ranging from 1 (extremely alert) to 10 (extremely sleepy).

#### 2.3.3 Procedure

The reading experiments, detailed in Section 2.3.1, followed the same procedure at both testing sites. Each participant completed a total of four experimental sessions, with approximately 1 week between any two sessions. Informed consent was obtained prior to the start of each session.

**Zurich**

In Zurich, the data were recorded at the Digital Linguistics Laboratory (DiLi Lab) at the Department of Computational Linguistics, University of Zurich. Two sessions were conducted with ET and two with SPR, and psychometric tests were always administered before the reading experiment. An overview of tasks conducted in each session is provided in Table 3. In the first ET session, participants completed the Mehrfachwahl‐Wortschatz‐Intelligenz test (MWT‐B, Lehrl, 2005), the first part of the Reynolds Intellectual Assessment Scales and Screening (RIAS, Hagmann‐von Arx & Grob, 2014), and the Lese‐ und Rechtschreib test (SLRT‐II, Moll & Landerl, 2010) before reading the four texts, while in the second ET session, the second part of RIAS was conducted before reading the four texts. In the first SPR session, participants completed the Frankfurter Aufmerksamkeits‐Inventar 2 (FAIR‐2, Moosbrugger, Oehlschlägel, & Steinwascher 2011), Stroop, and Simon tasks (Stroop, 1992) before the reading experiment, and in the second SPR session, they completed the working‐memory battery (WMC, Lewandowsky et al., 2010) before reading. On average, each reading experiment took 20–40 min.

**Table 3:** Tasks across sessions in Zurich: The order of sessions was randomized across participants with the restriction that *Eye‐tracking 1* was conducted before *Eye‐tracking 2* and *Self‐paced reading 1* was conducted before *Self‐paced reading 2*. The stimulus texts as well as the comprehension question types were counterbalanced across sessions

Method	Session	Tasks
Eye‐tracking	1	MWT‐B, RIAS, SLRT‐II, reading
Eye‐tracking	2	RIAS, reading
Self‐paced reading	1	FAIR‐2, Stroop, Simon, reading
Self‐paced reading	2	WMC, reading

**Potsdam**

The data were recorded in the eye‐tracking laboratory of the Department of Computer Science, University of Potsdam. Participants completed four eye‐tracking sessions, two of which were conducted under adverse conditions (see footnote 7) and are not included in our analyses. No self‐paced reading sessions were conducted. Alongside the reading experiments, participants also completed further tasks. The Psychomotor Vigilance Test (PVT, Dinges & Powell, 1985) was conducted twice, once before and once after reading, and the Jumping Dots Task, adopted from Makowski et al. (2021), was completed after the reading experiment.

### 2.4 Technical setup

#### 2.4.1 Eye‐tracking and self‐paced reading

ET data were collected with an Eyelink Portable Duo manufactured by SR Research mounted on a height‐adjustable desk in front of the presentation monitor (24 inch, screen size 43 cm × 31 cm, image resolution 1920×1080 px). The stimulus presentation window was set to 1280×1024 px in order to ensure optimal calibration. The remainder of the screen was colored in black. Binocular eye movements were recorded at a sampling rate of 2000 Hz. The eye‐to‐screen‐distance was 60 cm, and the eye‐to‐camera‐distance was 45 cm. A forehead‐ and chin‐rest was used in order to minimize participants' head movements. A keyboard for answering comprehension questions was placed between the participant and the camera so that participants were able to see the keyboard without moving their heads. Participants responded by pressing the “J” or “N” keys for yes/no questions and the “A,” “B,” “C,” or “D” keys for multiple‐choice questions. SPR data were collected on the same screen and with the same stimulus presentation window as in the ET sessions.

#### 2.4.2 Assessment of individual cognitive capacities

The Stroop and Simon tasks as well as the working memory test battery were administered in a computer‐based format using a 24‐inch ASUS VG248QG monitor (32 cm × 56 cm) with an image resolution of 1920×1080 px. A Cherry KC 1000 USB keyboard was used as a response device. All other tests were administered on paper following the exact procedure defined in the tests' manuals.

## 3 Methods and analyses

### 3.1 Preprocessing of ET data

We preprocessed the ET data using the following pipeline: We first converted the EyeLink Data Files to ASCII using the edf2asc tool from SR‐Research and then converted them to CSV with each row representing one sample including a timestamp and the x/y screen coordinates. We then extracted fixations and saccades using a data‐driven velocity‐based saccade detection algorithm (Engbert & Kliegl, 2003; Engbert, Sinn, Mergenthaler, & Trukenbrod, 2015).^12^ To account for vertical drift in the gaze recordings that resulted in fixations being mapped to the wrong lines of the stimulus text, we applied automatic fixation correction using dynamic time warping (Carr, Pescuma, Furlan, Ktori, & Crepaldi, 2021). Finally, we computed reading measures from fixations by mapping them to the white‐space delimited words. We adopted the terminology and definitions of reading measures used in Jakobi, Kern, Reich, Haller, and Jäger (2025). For our analyses, we used first‐fixation duration (FFD), first‐pass reading time (FPRT), RPD, TFT, first‐pass regression rate (FPReg), skips (SKIP), and number of fixations (N_FIX).^13^ We provide definitions in Table 4.

**Table 4:** Eye‐tracking reading measures that were used for the downstream analyses

Type	Measure name	Abbrev.	Timing	Description
Continuous	First fixation duration	FFD	First‐pass	Duration of the first fixation on a word.
	First‐pass reading time	FPRT	First‐pass	Sum of fixation durations during the first pass of a word before the reader moves forward or regresses.
	Regression‐path duration	RPD	Global	Sum of all fixation durations in the first pass of a word until fixating on a word to the right.
	Total fixation time	TFT	Global	Total duration of all fixations on a word.
Binary	First‐pass regression	FPReg	First‐pass	1 if a regression was initiated during the first pass of the word; otherwise, 0.
	Skipped word	SKIP	Global	1 if a word is never fixated; otherwise, 0.
Count	Number of fixations	N_FIX	Global	Total number of fixations on a word.

*Note*. Timing denotes the timing of the measures: *first‐pass* measures capture early processing during the initial encounter with a word, whereas *global measures* aggregate processing across both first‐ and later‐pass fixations (see Section 1.3.3).

Among these, FFD, FPRT, and FPReg are considered *first‐pass measures* reflecting cognitive processes that occur at early stages of language processing (e.g., lexical access), whereas the remaining measures are considered global measures (Boston, Hale, Kliegl, Patil, & Vasishth, 2008; Mézière et al., 2023), which also include later cognitive processes (e.g., syntactic reanalysis or repair).

### 3.2 Model‐based reliability analysis: Adapting the two‐task model

Rouder and Haaf (2019) demonstrated that non‐model‐based approaches where aggregated behavioral measures obtained from different conditions (e.g., mean TFT in a high‐surprisal vs. low‐surprisal condition) are directly correlated against one another underestimate effect sizes and, therefore, mask correlations (see Section 1.3.4). As a remedy, they recommend the use of hierarchical models, that is, (generalized) linear mixed‐effects models. Through regularization (Efron & Morris, 1977), they attribute some of the variability in individual‐level effects to trial noise, thus producing more accurate estimates.

Accordingly, in order to examine the reliability of individual differences in naturalistic reading across measurement occasions and across methods, we operationalized measurement reliability as the Pearson correlation between subject‐level effect estimates—the random intercepts and slopes—across two recording sessions or methods. Due to the linear nature of the Pearson correlation coefficient, correlating random‐effect estimates is mathematically equivalent to correlating the complete subject‐level effects that include the global (fixed‐effect) estimates.

Specifically, we adopted Rouder and Haaf's (2019) two‐task model, which has been successfully applied to study individual differences in nonlinguistic cognitive tasks (see, e.g., Veríssimo, Verhaeghen, Goldman, Weinstein, & Ullman, 2022; Rouder et al., 2023) and sentence processing (Frinsel & Christiansen, 2024; Staub, 2021). At its core, the model is a (generalized) linear‐mixed model with reaction time or another behavioral measure as the response variable. The variable whose reliability is to be assessed is used as a predictor both on the population level (i.e., as a fixed effect) and, crucially, at the individual level for each session or method separately (i.e., as a by‐subject‐by‐session random effect). Originally, Rouder and Haaf's (2019) two‐task model was developed to compare a single predictor—inhibitory cognitive control—across tasks. We extended this framework to multiple predictors in order to investigate the reliability of phenomena such as effects of lexical frequency, surprisal, word length, and dependency relations, which all play an important role for psycholinguistic theory building. Although these variables could be discretized as done by Staub (2021) for frequency and surprisal, doing so would require arbitrary binning decisions, reduce statistical power, and may eventually compromise the accuracy of estimating individual differences. Therefore, we adapted the two‐task model to fit multiple continuous variables simultaneously, extending its applicability to a wider range of reading phenomena and experimental designs. These changes are essential for paradigms such as naturalistic reading that require continuous predictors for its analyses.

#### 3.2.1 Model for the estimation of measurement reliability across measurement occasions

To estimate measurement reliability of the individual effects in response to the psycholinguistic predictors of interest (see below) across measurement occasions, we modeled a range of word‐level reading measures y: FFD, FPRT, RPD, TFT (continuous), FPReg and SKIP (binary), and N_FIX (count) for the ET data (see Table 4), and reaction times (continuous) for the SPR data, across two measurement occasions, using a linear‐mixed‐effects model. The predictor variables included word length, lexical frequency, surprisal, dependency distance, and the number of left dependents (see Section 2.2.2).

**Fig. 3:**
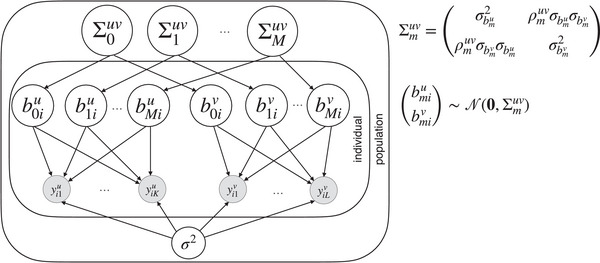
Random‐effects structure of the hierarchical correlation model for continuous response variables: Graphical representation of the hierarchical model for assessing measurement reliability of psycholinguistic predictors across measurement occasions for continuous response variables (FFD, FPRT, TFT, RPD, and SPR). Shaded nodes represent observed variables, and white nodes represent latent variables. For each subject i, we estimate individual effects b (random intercepts and slopes) for all M psycholinguistic predictor variables at two measurement occasions u and v. For a psycholinguistic predictor m∈{1,⋯,M} (e.g., word length), the correlation coefficient $\rho_{m}^{uv}$, contained in the covariance‐matrix $\Sigma_{m}^{uv}$, indicates the predictor's reliability across measurement occasions. $b_{0}$ denotes by‐subject‐by‐session random intercepts, and $y_{i1}^{s},y_{i2}^{s},\cdots$ denote all observations (i.e., eye‐tracking measures or self‐paced reading times) of subject i in session s, where s∈{u,v}, K being the total number of trials (i.e., words) in session u, and L being the total number of trials in session v. $\sigma^{2}$ refers to the residual variance (also called trial‐by‐trial variance or dispersion parameter). For clarity of presentation, fixed effects $\beta_{0},\cdots \beta_{M}$ are not included in this graph.

The correlation model simultaneously estimates population‐ and individual‐level effect sizes. Importantly, the model estimates separate individual‐level effects for each session. These individual‐level effects are, however, modeled jointly, allowing us to directly assess the correlation coefficients of individual effects from the variance‐covariance matrix. This is illustrated in the Bayesian Graphical Model of the random effect structure for the models with continuous target variables in Fig. 3. The full model is specified as follows:

(5) $$\ell \left(\theta_{i,j}\right)=\beta_{0}+b_{0,i}^{u}\delta_{j}^{u}+b_{0,i}^{v}\delta_{j}^{v}+\beta_{v}\delta_{j}^{v}+\sum_{m=1}^{M}\left(\beta_{m}+b_{m,i}^{u}\delta_{j}^{u}+b_{m,i}^{v}\delta_{j}^{v}\right)x_{m,j},$$

where $\theta_{i,j}$ denotes the distributional mean parameter for the *j* ^th^ observation (i.e., reading measure) y of participant i.^14^ The link function ℓ(·) specifies the transformation between the linear predictor and the distributional mean parameter $\theta_{i,j}$ and depends on the likelihood family of the dependent variable. A prediction ${\hat{y}}_{i,j}$ is obtained by sampling a value from the target variable's assumed distribution. For continuous reading time measures (SPR, FPRT, RPD_inc, TFT, FD), the likelihood family is lognormal, that is, $log\left(Y_{i,j}\right)∼\mathrm{Normal}\left(\mu_{i,j},\sigma^{2}\right)$, with the identity link, that is, $\mu_{i,j}=\theta_{i,j}$. For binary measures (FPReg, SKIP), the likelihood family is Bernoulli, $Y_{i,j}∼\mathrm{Bernoulli}\left(p_{i,j}\right)$, with the logit link, that is, $p_{i,j}=logit\left(\theta_{i,j}\right)$, where $p_{i,j}$ denotes the probability of a regression or a skip. For count measures (N_FIX), the likelihood family is zero‐inflated Poisson, $Y_{i,j}∼$ Zero‐Inflated Poisson $\left(\lambda_{i,j},\pi \right)$, with the log link for the Poisson mean parameter $\lambda_{i,j}$. Thus, $\lambda_{i,j}=log\left(\theta_{i,j}\right)$. The zero‐inflation probability π is modeled as an intercept‐only term with a logit link.^15^

On the right‐hand side of Eq. 5, $x_{m,j}$ denotes the z‐score normalized value of the m‐th psycholinguistic predictor variable for m=1,⋯,M. The global intercept is denoted by $\beta_{0}$, and $\beta_{v}$ refers to the session effect which models potential session‐related learning effects. It is coded via treatment contrast, that is, the effect represents the speed‐up (positive coefficient) or slow‐down (negative coefficient) in the dependent variable during the second session v compared to the first session u. $\delta_{j}$ are indicator functions $\delta_{j}^{u}=1\left[u=\text{session}\left(i,j\right)\right]$ and $\delta_{j}^{v}=1\left[v=\text{session}\left(i,j\right)\right]$, where session(i,j)∈{u,v} maps observation j of participant i to its session. $\beta_{m}$ represents the population‐level effect of the m‐th predictor, while $b_{m,i}^{\left\{u,v\right\}}$ are the random slopes for participant i in these sessions, which are the parameters of interest.^16^

Defined as such, the model is able to account for various sources of variability: The by‐subject‐by‐session random intercepts absorb potential subject‐specific, session‐related differences in reading times, irrespective of the predictors of interest; their variability is captured with $\sigma_{b_{0,i}^{\left\{u,v\right\}}}$, while $\sigma_{b_{m}^{\left\{u,v\right\}}}$ captures the between‐subjects variability of the effect sizes in response to the psycholinguistic predictor variables. Lastly, for continuous response variables (FFD, FPRT, TFT, RPD, and SPR), overall trial‐by‐trial noise is represented by the dispersion parameter σ which reflects the amount of unexplained variance in the log‐transformed response and governs the spread of the log‐normal distribution. As stated previously, $\sigma_{b_{m,i}^{u}}$ and $\sigma_{b_{m,i}^{v}}$ are contained in the covariance matrix $\Sigma_{m}^{uv}$, as well as the correlation coefficient $\rho_{m}^{uv}$ which we use as a measure of the reliability of the individual effect sizes $b_{m,i}^{u}$ and $b_{m,i}^{v}$ across sessions u and v, as illustrated in Fig. 3.

#### 3.2.2 Model for the estimation of cross‐method reliability

To assess cross‐method reliability, we fit an extended version of the model used to estimate reliability across measurement occasions that includes spillover effects (see below). Here, we correlate the effects of the psycholinguistic predictor variables *across methods*. To this end, we first combine the two sessions recorded with the same method. We then correlate SPR reaction times against a representative selection of ET reading measures (FPRT, RPD, TFT) that cover both first‐pass and global, as well as regression‐based measures. Thus, u and v do not represent two sessions of the same measure, but rather denote the two methods (measurement techniques) that are being correlated, namely, the ET measure of interest u and SPR times v, respectively. Note that in this context, the session effect $\beta_{v}$ captures potential overall differences in reading times between the respective ET measure and SPR. Since SPR and ET are known to induce different degrees of spillover effects, we additionally include random slopes for the psycholinguistic predictor variables of the previous word, formalized as follows:

(6) $$\begin{array}{ccc} \ell (\theta_{i,j}) & = & \beta_{0}+b_{0,i}^{u}\delta_{j}^{u}+b_{0,i}^{v}\delta_{j}^{v}+\beta_{v}\delta_{j}^{v}+\sum_{m=1}^{M}\left(\beta_{m}+b_{m,i}^{u}\delta_{j}^{u}+b_{m,i}^{v}\delta_{j}^{v}\right)x_{m,j}\\ & & +\;\left(b_{M+m,i}^{u}\delta_{j}^{u}+b_{M+m,i}^{v}\delta_{j}^{v}\right)x_{m,j-1}. \end{array}$$

Compared to the model defined in Eq. 5 that was used to examine measurement reliability across measurement occasions, Eq. 6 includes additional random slopes $b_{M+1,i}\cdots b_{M+M,i}$ that represent the effects of the psycholinguistic predictors of the previous word (i.e., $x_{m,j-1}$) on the reading time of the current word j, allowing us to study the correlation between individual effects induced by the current word (local effect) in ET measures with individual effects induced by the previous word (spillover effect) in SPR times.

#### 3.2.3 Model fitting

We fitted the models using brms (Bürkner, 2017) with four parallel chains, each running 2000 iterations, including 1000 warm‐up iterations. Model convergence was verified by examining the $\hat{R}$‐statistic and the trace plots (Gelman, Carlin, Stern, & Rubin, 1995). We report our priors for the different models specific to the three different types of dependent variables (continuous, binary, and count) in Table 5. They are mildly informative and in line with other studies that model reading time data (Nicenboim, Schad, & Vasishth, 2025; Wilcox, Ding, Sachan, & Jäger, 2024). For the models estimating reliability across measurement occasions in FFD and the ones estimating cross‐method reliability, we observed low convergence for a few parameters. We were able to fix these convergence issues by increasing the iterations to 4000 (including 2000 warm‐up) and increasing the priors' precision. For these models only, we, therefore, set $\beta_{0}∼\mathrm{Normal}\left(5.5,0.1\right)$, translating to an expected grand mean reading time of exp(5.5)≈ 245 ms with a 95% RTs range of approximately 200–300 ms. We further defined $\beta_{m}$’s prior as Normal(0,0.1), assuming that one unit change in predictors results in an RT change of about 25 ms.

**Table 5:** Overview of priors defined for the hierarchical models defined in Eqs. 5 and 6

Continuous	Binary	Count
$\beta_{0}∼\mathrm{Normal}\left(6,1\right)$	$\beta_{0}∼\mathrm{Normal}\left(0,0.1\right)$	$\beta_{0}∼\mathrm{Normal}\left(0,0.1\right)$
$\beta_{m}∼\mathrm{Normal}\left(0,1\right)$	$\beta_{m}∼\mathrm{Normal}\left(0,0.5\right)$	$\beta_{m}∼\mathrm{Normal}\left(0,0.5\right)$
$sd\left(b_{m}\right)∼\mathrm{Exponential}\left(2\right)$	$sd\left(b_{m}\right)∼\mathrm{Exponential}\left(2\right)$	$sd\left(b_{m}\right)∼\mathrm{Exponential}\left(2\right)$
$\Omega_{m}^{uv}∼\mathrm{LKJ}\left(2\right)$	$\Omega_{m}^{uv}∼\mathrm{LKJ}\left(2\right)$	$\Omega_{m}^{uv}∼\mathrm{LKJ}\left(2\right)$
$\sigma_{m}∼\mathrm{Exponential}\left(2\right)$		

*Note*. LKJ denotes the Lewandowski–Kurowicka–Joe distribution. Note that $\Omega_{m}^{uv}$ is the correlation matrix decomposed from $\Sigma_{m}^{uv}$ (see Fig. 3) such that $\Sigma_{m}^{uv}=S_{m}^{uv}\Omega_{m}^{uv}S_{m}^{uv}$, where $S_{m}^{uv}=\sqrt{\mathrm{diag}\;(\Sigma_{m}^{uv})}$.

## 4 Results

In the following, we first present the results of the model assessing measurement reliability across measurement occasions in Section 4.1, including the fixed‐effects estimates (Section 4.1.1) and the reliability estimates (Section 4.1.2). We then report the cross‐method reliability results in Section 4.2. Finally, we examine the ratio of true individual‐level effect variability and trial‐by‐trial variability in Section 4.3.

### 4.1 Measurement reliability of individual differences across measurement occasions

#### 4.1.1 Population‐level effects of psycholinguistic predictors

**Fig. 4:**
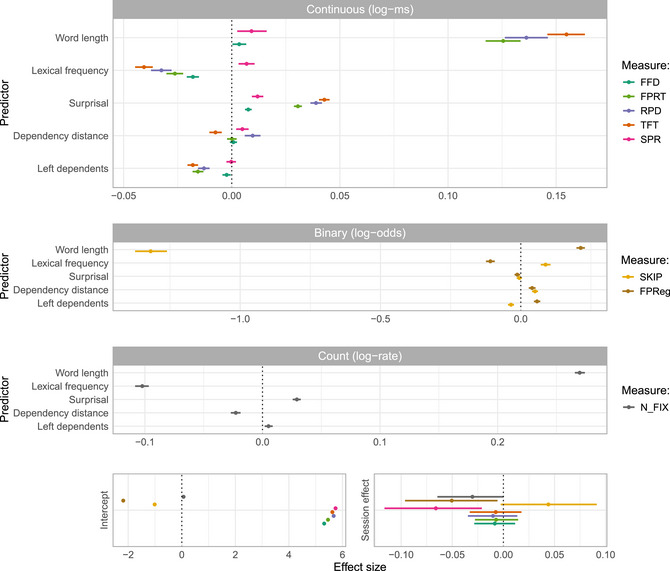
Population‐level effect estimates: Population‐level posterior distributions for all psycholinguistic predictor variables, the intercept (bottom left panel) and the session effect (bottom right panel) observed in different eye‐tracking measures: first‐fixation duration (FFD), first‐pass reading time (FPRT), total fixation time (TFT), regression‐path duration (RPD), skipped words (SKIP), first‐pass regressions (FPReg), and number of fixations (N_FIX) as well as self‐paced reading times (SPR). Effect sizes are in log space (natural logarithms). We report the posterior means together with a 95% credible interval for each reading measure.

We first provide an overview of the population‐level effect estimates. Fig. 4 displays the posterior distributions for all predictors across eight dependent measures, seven ET reading measures—FFD, FPRT, RPD, TFT, SKIP, and FPReg, N_FIX—as well as SPR.

**Intercept**: The global intercept coefficient represents population‐level reading patterns across participants depending on the reading measure. As an illustration, the intercept coefficient may represent, for example, the grand mean first‐pass reading time, or the grand mean number of total fixations. The magnitudes of the coefficients were within the expected ranges, and the reading times showed positive coefficients, ranging from 5.3 log‐ms in FFD to 5.7 log‐ms in SPR, translating to average reading times of approximately 200–300 ms per word. For the binary measures, the intercept represents the average log‐odds of first‐pass regressions and skips, respectively. They can be back‐transformed to probabilities using the inverse logit function, resulting in average probabilities of $logit^{-1}\left(-2.43\right)\approx 0.08$ for FPReg and $logit^{-1}\left(e^{-0.41}\right)\approx 0.40$ for SKIP.

**Session effect**: The bottom right panel of Fig. 4 shows the session effect, that is, the overall difference of a given reading measure between the first and second sessions. A negative coefficient of the continuous reading measures implies that readers exhibited shorter reading times. Readers exhibited shorter reading times during the second SPR session. They also showed fewer regressions in the second compared to the first ET session. For SKIP, there was a tendency toward more skips in the second ET session, although the 95% CrI narrowly included 0, making this result inconclusive. For all other ET measures, 0 lay well within the 95% CrI, providing no conclusive evidence for a session effect.

**Word length**: As expected, we found that longer words led to more fixations, more regressions, and generally longer reading times across all ET measures, as well as longer SPR times. The strong negative effect on SKIP (−1.32 [−1.37, −1.26]) indicates that readers were less likely to skip longer words.

**Lexical frequency**: Using log‐lemma frequency as a predictor, we found that higher‐frequency words were associated with more skips and fewer fixations and regressions, and shorter reading times across all ET measures (FFD, FPRT, RPD, and TFT). In contrast, in SPR, higher‐frequency words elicited longer reading times.

**Surprisal**: For surprisal, the results indicate that higher surprisal consistently increased reading times across all continuous ET measures as well as SPR time, confirming that unexpected words impose higher processing load (Hale, 2001; Levy, 2008). Moreover, words with high surprisal led to longer RPD. The effects of surprisal on the binary measures were either very small (FPReg) or inconclusive (SKIP).

**Dependency distance**: Turning to the syntactic predictors, we observed a locality effect in head‐initial constructions: a longer distance between a dependent and its head led to more regressions (FPReg) initiated from this dependent, resulting in longer RPD and SPR times.

In contrast, readers showed fewer fixations and more skips on the dependent itself, which yielded shorter total reading times (TFT), an effect that can be interpreted as an *anti*‐locality effect. The impact of dependency distance on FFD and FPRT was inconclusive, with posterior means being close to zero in both cases.

**Number of left dependents**: The effect of the number of left dependents again yielded a mixed picture. On the one hand, heads with more left dependents elicited more regressions from them, more fixations, and fewer skips. On the other hand, reading times across all continuous ET measures were longer for heads with more left dependents. As before, the results for SPR times were inconclusive.

#### 4.1.2 Measurement reliability

**Fig. 5:**
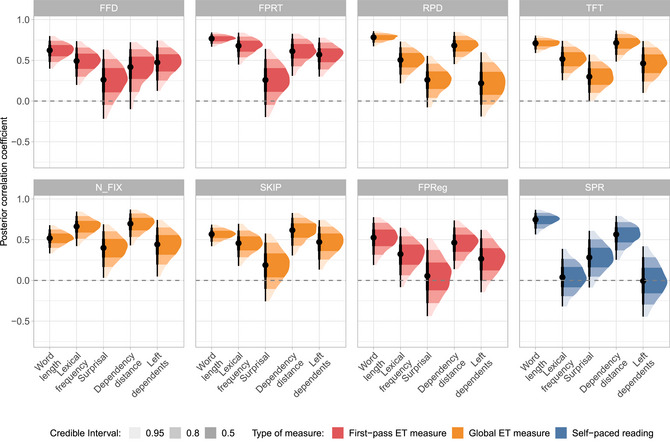
Correlation coefficients for measurement reliability across experimental sessions: Posterior distributions of the correlation coefficients across sessions for the individual effects in response to the psycholinguistic predictors based on reading measures from the eye‐tracking and SPR data. For each distribution, the 50‐, 80‐, and 90% credible intervals are shown, indicated by the transparency of the shading.

Next, we present the reliability estimates of the individual‐level psycholinguistic predictors between two measurement occasions, operationalized by the correlation coefficients contained in the random‐effects covariance matrix. Posterior distributions are shown in Fig. 5 and Table 6.^17^

**Table 6:** Measurement reliability of psycholinguistic effects across experimental sessions: Posterior distributions of the correlation coefficients for measurement reliability across experimental sessions of different psycholinguistic predictors and reading measures from the ET and SPR data

Measure	Intercept	Word length	Lexical freq.	Surprisal	Dependency dis.	Num. left dep.
FFD	**0.73** [0.63, 0.80]	**0.62** [0.40, 0.80]	**0.48** [0.20, 0.74]	0.25 [−0.22, 0.63]	0.39 [−0.10, 0.72]	**0.46** [0.12, 0.74]
FPRT	**0.73** [0.65, 0.81]	**0.76** [0.67, 0.84]	**0.67** [0.45, 0.84]	0.25 [−0.20, 0.64]	**0.60** [0.31, 0.83]	**0.56** [0.30, 0.78]
RPD	**0.74** [0.66, 0.81]	**0.78** [0.67, 0.86]	**0.50** [0.22, 0.73]	0.25 [−0.08, 0.55]	**0.67** [0.45, 0.85]	0.22 [−0.19, 0.60]
TFT	**0.72** [0.63, 0.79]	**0.71** [0.59, 0.80]	**0.51** [0.26, 0.74]	**0.30** [0.01, 0.57]	**0.70** [0.48, 0.87]	**0.45** [0.10, 0.74]
N_FIX	**0.80** [0.72, 0.86]	**0.51** [0.33, 0.68]	**0.66** [0.42, 0.84]	**0.39** [0.03, 0.69]	**0.68** [0.43, 0.87]	**0.43** [0.05, 0.74]
SKIP	**0.77** [0.70, 0.83]	**0.56** [0.42, 0.69]	**0.45** [0.18, 0.69]	0.18 [−0.26, 0.58]	**0.60** [0.31, 0.83]	**0.46** [0.13, 0.74]
FPReg	**0.86** [0.81, 0.91]	**0.52** [0.19, 0.78]	0.31 [−0.08, 0.65]	0.05 [−0.44, 0.52]	**0.45** [0.14, 0.74]	0.26 [−0.15, 0.62]
SPR	**0.78** [0.67, 0.86]	**0.74** [0.56, 0.87]	0.04 [−0.32, 0.39]	0.28 [−0.09, 0.61]	**0.55** [0.25, 0.79]	−0.01 [−0.44, 0.42]

*Note*. We present the mean estimates and the 95% credible intervals. Bold font denotes that 0 is not included in the 95% credible interval.

**Intercept**: The random intercept coefficients showed strong measurement reliability across experimental sessions for all reading measures, with posterior correlation estimate means ranging from 0.72 (TFT) to 0.86 (FPReg). High reliability estimates were observed for number of fixations and skips, which both reflect broader, more general reading patterns. This suggests that general individual reading strategies were particularly stable over time.

**Word length**: The word length effect exhibited robust measurement reliability across experimental sessions, particularly when assessed with first‐pass and global measures. For RPD, the correlation coefficient was 0.78 [0.67, 0.86] and 0.76 [0.67, 0.84] for FPRT, indicating that individual sensitivity to word length was reliable between experimental sessions during early‐stage and global processing. TFT and SPR also demonstrated strong reliability ($\hat{\rho }=0.71$, [0.59, 0.80] for TFT and $\hat{\rho }=0.74$, [0.56, 0.87] for SPR). Other ET measures were associated with lower correlation coefficients ($\hat{\rho }\leq 0.62$), but still showed moderate measurement reliability across experimental sessions, with all 95% CrIs excluding zero.

**Lexical frequency**: The lexical frequency effect exhibited at best moderate reliability across most reading measures, except for SPR ($\hat{\rho }=0.04$, [−0.32, 0.39]), the correlation coefficient being close to zero. The highest measurement reliability across measurement occasions was observed in FPRT, a first‐pass measure ($\hat{\rho }=0.67$, [0.45, 0.84]), followed by fixation count (N_FIX; $\hat{\rho }=0.66$, [0.42, 0.84]). When assessed with the other count‐based measure, SKIP, lexical frequency exhibited lower reliability ($\hat{\rho }=0.45$, [0.18, 0.69]). Last, lexical frequency showed poor measurement reliability across measurement occasions when assessed with first‐pass regressions ($\hat{\rho }=0.31$, [−0.08, 0.65]) and SPR ($\hat{\rho }=0.04$, [−0.32, 0.39]), with wide credible intervals including zero, indicating that participants' early regression strategies in response to varying lexical frequencies were inconsistent across sessions.

**Surprisal**: Individual surprisal effects showed poor measurement reliability across measurement occasions. The highest correlation coefficients were associated with fixation count (N_FIX, $\hat{\rho }=0.39$, [0.03, 0.69]) and TFT ($\hat{\rho }=0.30$, [0.01, 0.57]), which are both global measures. For all other ET measures as well as SPR times, the results were inconclusive with the 95% CrI including zero, indicating that the reliability of individual‐level surprisal effects was poor in these measures.

**Dependency distance**: The dependency distance effect showed moderate measurement reliability among participants across global reading measures. In RPD, the estimated correlation coefficient was 0.67, [0.45, 0.85], while in TFT, it reached 0.70, [0.48, 0.87]. When assessed in first‐pass measures or SPR, reliability across measurement occasions was lower: $\hat{\rho }=0.60$, [0.31, 0.83] for FPRT, $\hat{\rho }=0.55$, [0.25, 0.79] for SPR, $\hat{\rho }=0.45$, [0.14, 0.74] for FPReg, and inconclusive for FFD ($\hat{\rho }=0.39$ [−0.10, 0.72]).

**Number of left dependents**: The observed effects of the number of left dependents showed moderate reliability in the first‐pass reading measure FPRT ($\hat{\rho }=0.56$, [0.30, 0.78]). Reliability estimates were lower in TFT and N_FIX, with correlation coefficients of 0.45 [0.10, 0.74] and 0.43 [0.05, 0.74], respectively. A similar degree of reliability was found for the effect of the number of left dependents on skips ($\hat{\rho }=0.46$, [0.13, 0.74]). Participants' sensitivity to the number of left dependents was not found to be reliable across measurement occasions when measured with first‐pass fixations (FFD), with a posterior correlation coefficient of 0.46 [0.12, 0.74]). For the remaining measures, the results were inconclusive, with the mean posterior correlation coefficient being 0.22 (RPD), 0.26 (FPReg), and −0.01 (SPR).

Overall, the reliability of individual‐level differences in reading behavior across measurement occasions was robust with respect to the random intercept and the word length effect. This result was consistent across all tested reading measures. Dependency distance showed moderate measurement reliability, except when measured with first‐pass measures. Lexical frequency, surprisal, and the number of left dependents exhibited low to poor reliability, with only lexical frequency approaching moderate reliability when assessed with the global count‐based measure (N_FIX) or first‐pass reading time (FPRT). As a concluding observation, we noted that measurement reliability was generally higher for ET measures of reading than for SPR data.

### 4.2 Cross‐method reliability of individual differences

Next, we assess the cross‐method reliability of individual‐level differences in reading between ET and SPR measures using data from 60 participants tested in Zurich who completed all four sessions. Analogous to the analyses assessing measurement reliability across measurement occasions, cross‐method reliability is operationalized as the Pearson correlation coefficient obtained from the hierarchical model described in Section 3.2.2, fitted on data combining continuous ET measures (FPRT, RPD, or TFT) and SPR times. RPD and TFT were selected for their similarity to SPR times, while FPRT, the most commonly used first‐pass measure, was included as an additional point of comparison. In addition to local effects (i.e., effects from the current word's psycholinguistic predictor variable on that word), the model also included spillover effects (i.e., effects from the previous word's psycholinguistic predictor variable on the current word).

**Fig. 6:**
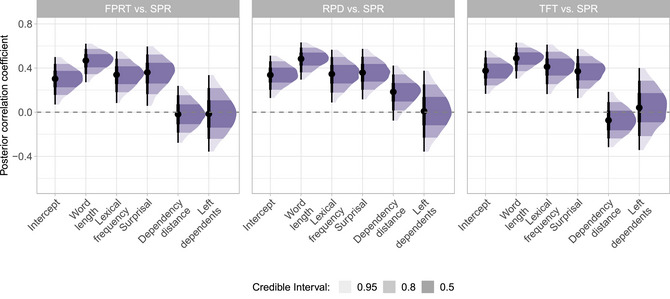
Correlation coefficients for cross‐method reliability: Posterior distributions of the Pearson correlation coefficients for cross‐method reliability of the different psycholinguistic predictors and the intercept. For each of these predictor variables, correlations are computed between the by‐subject random effects obtained from one of the eye‐tracking measures (first‐pass reading time [FPRT], regression‐path duration [RPD], total fixation time [TFT]) and the by‐subject random effects obtained from the SPR times. For all coefficients except the intercept, we report the correlation between the effect of the current word's predictor variable in eye‐tracking and the effect of the *previous* word's predictor in SPR (i.e., the “spillover”). For each posterior distribution, the 50‐, 80‐, and 95% credible intervals are shown.

Overall, we observed poor cross‐method reliability in terms of correlation coefficients between the individual‐level *local* effects in ET reading times and the individual‐level *local* effects in SPR times, as demonstrated in Fig. D.2 in the Appendix. However, when examining correlations between individual‐level *local* effects in ET reading times and the individual‐level *spillover* effects in SPR times, considerably higher reliability estimates emerged for word length, lexical frequency, and surprisal effects. For this reason, the following results on cross‐method reliability are reported based on the SPR spillover effect (see Fig. 6 and Table 7). Unlike the previous analysis of measurement reliability across measurement occasions, we do not summarize the population‐level effects. These can be found in Appendix D.2. In the cross‐method model, fixed effects are estimated jointly across both methods, which makes them difficult to interpret.

**Table 7:** Cross‐method reliability of psycholinguistic effects: Posterior distributions of the Pearson correlation coefficients for the cross‐method reliability of different psycholinguistic predictors measured with eye‐tracking (FPRT, RPD, or TFT) in comparison to SPR times

Measures	Intercept	Word length	Lexical freq.	Surprisal	Dependency dis.	Num. left dep.
FPRT‐SPR	**0.30** [0.07, 0.50]	**0.46** [0.27, 0.62]	**0.33** [0.08, 0.55]	**0.35** [0.06, 0.60]	−0.02 [−0.28, 0.24]	−0.01 [−0.36, 0.34]
RPD‐SPR	**0.33** [0.13, 0.51]	**0.48** [0.30, 0.63]	**0.34** [0.09, 0.57]	**0.35** [0.12, 0.57]	0.18 [−0.08, 0.42]	0.01 [−0.36, 0.38]
TFT‐SPR	**0.37** [0.17, 0.56]	**0.48** [0.31, 0.63]	**0.41** [0.16, 0.61]	**0.36** [0.13, 0.57]	−0.07 [−0.32, 0.18]	0.04 [−0.34, 0.40]

*Note*. Reliability estimates are based on SPR spillover effects (see Section 4.2 for details). We present the mean estimates and the 95% credible intervals. Bold font denotes that 0 is not included in the 95% credible interval.

**Intercept**: The by‐subject‐by‐session random intercepts showed low to moderate cross‐method reliability between ET and SPR measures, with the highest reliability observed in TFT‐SPR ($\hat{\rho }=0.37$, [0.17, 0.56]), followed by RPD‐SPR ($\hat{\rho }=0.33$, [0.13, 0.51]), and FPRT‐SPR ($\hat{\rho }=0.30$, [0.07, 0.50]). These results indicate that individual‐level differences in overall reading speed were not consistent across methods: a participant who read quickly in an ET session did not necessarily read quickly in an SPR session.

**Word length**: Word length effects exhibited the highest posterior correlation coefficients among all psycholinguistic predictors, regardless of which ET measure was compared to SPR times. Mean correlation coefficients ranged from 0.46 (FPRT‐SPR) to 0.48 (RPD‐SPR and TFT‐SPR), and the 95% CrIs largely overlapped, indicating that the type of ET measure did not affect the cross‐method reliability estimate.

**Lexical frequency**: Cross‐method reliability for individual effects of lexical frequency was generally lower than for word length. The highest reliability was observed for TFT‐SPR times ($\hat{\rho }=0.41$, [0.16, 0.61]), followed by slightly lower values for RPD‐SPR ($\hat{\rho }=0.34$, [0.09, 0.57]) and FPRT‐SPR ($\hat{\rho }=0.33$, [0.08, 0.55]).

**Surprisal**: Surprisal showed reliability comparable to lexical frequency. The mean posterior correlation coefficients were similar across the different ET measures, with $\hat{\rho }=0.35$ (FPRT‐SPR, RPD‐SPR) and $\hat{\rho }=0.36$ (TFT‐SPR), respectively.

**Dependency distance**: Dependency distance showed poor cross‐method reliability and high uncertainty, as indicated by wide CrIs across all comparisons. This suggests that individual‐level effects of longer distances between a dependent and its head observed with one method did not replicate in the other method.

**Number of left dependents**: Similarly, correlations between readers' sensitivity to the number of left dependents in ET and SPR were very small. For all ET measures, posterior means were close to zero and 95% CrIs are wide, indicating generally low measurement reliability across methods.

### 4.3 Estimating the ratio between true individual variability and trial‐by‐trial variability

To better contextualize the results of our reliability analyses, we now examine the estimates of the model's underlying sources of variance. Using hierarchical models allows us to separate trial‐by‐trial variability^18^ from the variability of the individual‐level effect estimates. As pointed out in Section 1.2, the larger the ratio of trial‐by‐trial variability to the true between‐subjects variability, the larger the expected attenuation of the observed correlation coefficient. Following Rouder et al. (2023), we report the estimated residual standard deviation $\hat{\sigma }=\sqrt{{\hat{\sigma }}^{2}}$, the estimated standard deviations across subjects of the individual‐level parameters (by‐subject‐by‐session random intercepts and slopes), ${\hat{\sigma }}_{m}=\sqrt{{\hat{\sigma }}_{m}^{2}}$, as well as the corresponding ratio of the individual‐level effect standard deviation to trial‐by‐trial standard deviation, referred to as signal‐to‐noise ratio (SNR) ${\hat{\gamma }}_{m}\overset{def}{=}\frac{{\hat{\sigma }}_{m}}{\hat{\sigma }}$ for all models with continuous response variables in Table 8.^19^

**Table 8:** Overview of residual standard deviation ($\hat{\sigma }$) and between‐subjects standard deviation of individual effects of psycholinguistic predictors ${\hat{\sigma }}_{m}$ for each session

Measure	Parameters													Sample sizes	
	$\hat{\sigma }$	${\hat{\sigma }}_{ri}$	${\hat{\gamma }}_{ri}$	${\hat{\sigma }}_{wl}$	${\hat{\gamma }}_{wl}$	${\hat{\sigma }}_{f}$	${\hat{\gamma }}_{f}$	${\hat{\sigma }}_{s}$	${\hat{\gamma }}_{s}$	${\hat{\sigma }}_{d}$	${\hat{\gamma }}_{d}$	${\hat{\sigma }}_{nl}$	${\hat{\gamma }}_{nl}$	$n_{obs}$	$n_{ind}$
FFD S1	.41	.14	.33	.02	.04	.01	.04	.00	.01	.00	.01	.01	.02	444,760	131
FFD S2	.41	.18	.44	.02	.04	.01	.03	.01	.01	.01	.02	.01	.02	444,760	131
FPRT S1	.44	.15	.35	.05	.11	.02	.05	.01	.02	.01	.02	.01	.03	384,644	131
FPRT S2	.44	.19	.44	.05	.11	.02	.04	.01	.01	.01	.02	.01	.03	384,644	131
RPD S1	.55	.18	.33	.06	.11	.03	.05	.02	.03	.02	.03	.01	.02	383,639	131
RPD S2	.55	.22	.40	.06	.10	.02	.04	.01	.02	.02	.03	.01	.02	383,639	131
TFT S1	.50	.17	.34	.05	.11	.02	.05	.02	.03	.01	.02	.01	.02	443,541	131
TFT S2	.50	.22	.44	.05	.10	.02	.04	.01	.02	.02	.03	.01	.02	443,541	131
SPR S1	.36	.28	.79	.03	.10	.02	.04	.01	.03	.01	.04	.01	.02	287,843	65
SPR S2	.36	.23	.64	.02	.07	.01	.03	.01	.02	.01	.02	.01	.02	287,843	65
FPRT	.39	.18	.46	.10	.26	.03	.08	.01	.03	.00	.01	.01	.03	442,359	60
SPR	.39	.28	.70	.03	.09	.01	.03	.01	.03	.01	.01	.00	.01	442,359	60
RPD	.44	.21	.48	.12	.26	.03	.08	.03	.06	.01	.01	.02	.04	442,681	60
SPR	.44	.27	.61	.03	.08	.01	.03	.01	.02	.00	.01	.00	.01	442,681	60
TFT	.42	.21	.49	.11	.27	.04	.08	.02	.05	.01	.01	.02	.04	465,292	60
SPR	.42	.27	.65	.03	.08	.01	.03	.01	.03	.01	.01	.00	.01	465,292	60

*Note*. The ratio ${\hat{\gamma }}_{m}$ = $\frac{{\hat{\sigma }}_{m}}{\hat{\sigma }}$ is also reported. Subscripts indicate the random intercept (ri) and the psycholinguistic predictors: word length (wl), lexical frequency (f), surprisal (s), dependency length (d), and number of left dependents (nl). $n_{obs}$ denotes the total number of data points used for model fitting, and $n_{ind}$ denotes the number of individuals. We only report results for models fitted on continuous response variables. For each reading measure, the first row corresponds to the first session, and the second row corresponds to the second session. For models fitted on cross‐method data, the first row reports statistics for the eye‐tracking sessions, and the second row reports for the SPR sessions. Note that for SPR sessions, the metrics are based on the spillover effects (see Section 4.2).

Let us first consider the models assessing measurement reliability across measurement occasions. An inspection of the variance components showed that the residual standard deviation $\hat{\sigma }$ was generally higher for ET data—in particular for RPD ($\hat{\sigma }=0.55$) and TFT ($\hat{\sigma }=0.50$)—than for SPR data ($\hat{\sigma }=0.36$). Considering SNRs, we note that $\hat{\gamma }$ for the by‐subject random intercepts is rather high across all measures with $\hat{\gamma }\geq 0.3$, suggesting that the expected attenuation of the correlation coefficient was low. Next, low SNR (i.e., $\hat{\gamma }\leq 0.11$) was observed for the individual word‐length effect. Even lower SNRs (i.e, $\hat{\gamma }\leq 0.04$) were observed for the remaining psycholinguistic predictors, suggesting a rather strong attenuation of the correlation coefficient.

In the cross‐method setting, the overall residual standard deviation $\hat{\sigma }$ ranged from 0.39 (FPRT) to 0.44 (RPD), which is comparable to the values observed for the models fitted across measurement occasions. The $\hat{\gamma }$ ratios were similar to those reported for models fitted separately on ET or SPR data. We observed slightly lower SNRs for individual surprisal and lexical frequency spillover effects across SPR sessions.

## 5 Discussion

In the following, we first discuss the results of the models assessing measurement reliability across measurement occasions and methods in Section 5.1. Then, we revisit the reliability paradox in the light of our empirical results in Section 5.2 before addressing some methodological considerations in Section 5.3. Finally, we discuss the implications of our work for theories of sentence processing in Section 5.4.

### 5.1 Model analyses

#### 5.1.1 Individuals' reading behavior and their sensitivity to word length are relatively stable between multiple measurement occasions

Our measurement reliability analysis across measurement occasions showed that on the individual level, both an individual's overall reading patterns captured by the random intercept term as well as their sensitivity to word length are relatively stable across experimental sessions of the same method—both within ET‐based measures and SPR reaction times.

The measurement reliability of the *by‐subject intercept* indicates that participants exhibited consistent individual reading patterns as reflected in fixation durations, first‐pass regressions, skip proportions, and total fixation counts in ET, and average reaction times in SPR. These individual reading patterns are likely not strongly influenced by fluctuations in an individual's state‐of‐mind on a given day, suggesting that they reflect deeply ingrained characteristics of an individual's reading behavior. This is in line with observations made by other eye‐tracking‐while‐reading studies that assessed the reliability of saccadic events and reading measures across different measurement occasions (Carter and Luke, 2018; Henderson & Luke, 2014), as well as studies showing that individual reading patterns can be used as a behavioral biometrics for identifying readers (Makowski, Jäger, Abdelwahab, Landwehr, & Scheffer, 2018).

With regard to the *word length* effect, we observed that an individual who is particularly sensitive to word length (i.e., showing a strong slowdown caused by a long word) on one day shows similar sensitivity when tested again on another day. This finding is likely related to the nature of the word length effect. From a neural processing perspective, longer words impose a larger processing load on the visual system (Schuster, Hawelka, Hutzler, Kronbichler, & Richlan, 2016). Given that visual processing presumably takes place at an earlier stage compared to higher‐level linguistic processes such as syntactic integration or lexical access, the stability of the individual word length effect might reflect the characteristics behind an individual's visual processing and lower‐level oculomotor control. The word length showed particularly high reliability in later measures such as RPD. Why might this be the case? The robust *fixed* effect estimate of the word length predictor in RPD suggests that the impact of word length is not limited to early visual or oculomotor processing, but extends into later stages of reading. Since higher RPDs reflect prolonged time spent revisiting earlier parts of the sentence, one plausible explanation is that longer words, simply by requiring more time to read, increase the risk of memory decay for earlier parts of the sentence. This temporal delay can disrupt the ongoing syntactic parsing process, making it more likely that readers lose track of structural dependencies and need to revisit previous content to re‐establish coherence. Turning to the *individual‐level* word length effect, the high reliability estimate in RPD suggests that the tendency of how strongly a given reader is disrupted by long words during integration is a stable trait.

#### 5.1.2 Reliability of individual higher‐level cognitive effects across measurement occasions strongly depends on the reading measure it is assessed on

Individual differences in *lexical frequency* effects are generally less reliable than those in the word length effect, both across measurement occasions and methods. This finding contrasts with Carter and Luke (2018), who observed high stability of individual frequency effects across measurement occasions. However, as noted by Staub (2021), frequency, word length, and predictability are highly intercorrelated in natural texts, making it challenging to isolate the unique effect of frequency. The models used by Carter and Luke (2018) assessed these effects separately, potentially conflating the contributions of all three variables due to their correlations (Brysbaert, Stevens, Mandera, & Keuleers, 2016). By choosing an approach where we model all predictor variables simultaneously, we obtain more conservative estimates. Moreover, we observed that reliability of the frequency effect was higher in some reading measures than in others. Specifically, we observed the highest measurement reliability across experimental sessions when it was assessed with total fixation count or first‐pass reading time. Conversely, reliability of the frequency effect between experimental sessions was particularly low when assessed in first‐pass regressions. This observation is in line with previous findings from Abbott and Staub (2015) and Staub (2021).

*Surprisal* is a strong predictor of reading times at the population level, as established by previous work (Hale, 2001; Levy & Keller, 2013; Smith & Levy, 2013, inter alia). When encountering high‐surprisal words, readers exhibited longer fixation times, are more likely to initiate regressions, and are less likely to skip these words. However, the reliability estimate for individual differences across measurement occasions is poor overall, at most moderate in global ET measures that include both first‐ and later‐pass readings such as TFT and N_FIX. Overall, one potential reason for the observed low to moderate reliability of individual‐level effects of surprisal is that corpus‐based estimates of surprisal may not fully capture the expectations of individual readers. Surprisal values are computed on the basis of language models trained on large corpora, which may not accurately reflect the linguistic experience of any individual. As a result, surprisal estimates may diverge from the readers' internal prediction model. This concern is supported by recent findings from Haller et al. (2024), who showed that the predictive power of language model‐based surprisal estimates is modulated by individual cognitive capacities, suggesting that the estimated surprisal values align better with the some readers' internal models of prediction than others'. A possible explanation for the difference in reliability between first‐pass and global measures is that, while predictive mechanisms are assumed to operate incrementally and early during processing, their behavioral consequences may accumulate and stabilize only at later stages where readers may verify whether the word they recognized aligns with their contextual predictions (reflected in TFT and N_FIX), or revisit the previous context to adjust their predictions (reflected in RPD). This leads to the individual sensitivity to surprisal in global measures being more stable as opposed to first‐pass measures. This highlights the complexity of surprisal effects: while first‐pass measures do not capture reliable individual characteristics, measures of later processing stages reflect more stable, trait‐like cognitive strategies that readers employ to resolve surprisal‐related difficulties.

#### 5.1.3 Dependency distance is the more reliable syntactic predictor than the number of left dependents

At the population level, we observed no effect of *dependency distance* (the distance between the currently read syntactic dependent and its head) in first‐pass ET‐based measures. By contrast, in RPD, readers are more likely to regress to earlier words during their first pass and spend more time revisiting previous material when encountering a dependent whose head is further away to help integrate the currently read dependent into its context (Bartek, Lewis, Vasishth, & Smith, 2011; Grodner & Gibson, 2005). At the same time, readers tend to fixate less and skip more, which is also reflected by shorter TFTs. This reveals a strategic contrast in how readers handle high‐surprisal words versus long‐dependency constructions. For high‐surprisal words, readers make more and overall longer fixations on the words themselves, trying to integrate the unexpected continuation into its context. In contrast, when encountering a dependent remotely from its (already encountered) syntactic head, readers often skip ahead to the upcoming context to aid integration, or regress back to previous material.

Regarding the individual‐level effect of dependency distance, we found its reliability across measurement occasions to be highest in global ET measures. In SPR, it represents the only higher‐level effect (i.e., besides the intercept and word length) that showed moderate measurement reliability across sessions. This result might arise from SPR being less naturalistic than eye‐tracking‐while‐reading. Pressing the space bar in SPR is a more conscious act than moving the gaze over a stimulus, and may prompt some readers to adjust their reading strategy to cope with individual task demands when confronted with a long‐distance dependency, for example, pausing to skip back and resolve the long‐distance dependency, but not others. Importantly, the results suggest that during SPR, readers will adopt the same behavior or strategy when tested again, reflected by the relatively high reliability across experimental sessions. However, the adopted individual strategy is not aligned with the one deployed in naturalistic reading recorded with ET, resulting in low cross‐method reliability of the dependency‐distance effect (see Section 5.1.4 for the general discussion on the cross‐method reliability results). Thus, taken together, these findings may not reflect genuine differences in sentence processing per se, but rather reader‐specific strategies for coping with nonlocal dependencies in the SPR setting. In future research, it would be worthwhile to expand our analyses to different types of SPR paradigms, such as the noncumulative moving‐window paradigm, where only one word at a time is displayed and readers do not have the possibility to revisit previous sections, or BSPR where readers are able to regress to earlier words or regions (Paape & Vasishth, 2022). Using these paradigms would help determine whether the ability to regress in the cumulative‐window SPR is a key factor driving the observed pattern of within‐method reliability.

The *number of left dependents* refers to the number of syntactic dependents preceding a head. At the population level, heads with more left dependents tended to show shorter reading times, presumably because these dependents increase predictability and, therefore, lead to faster processing of the head itself, often referred to as anti‐locality effect (Demberg & Keller, 2008; Levy, 2008; Vasishth & Lewis, 2006). However, despite this time efficiency, readers still exhibited more total fixations on these heads, skipped them less often, and initiated more regressions from them. Despite the overall facilitation in processing, the syntactic complexity introduced by the increased number of dependents likely demands additional attention to ensure proper integration and understanding of the sentence structure (Engbert, Nuthmann, Richter, & Kliegl, 2005). Readers may regress to earlier parts of the sentence to confirm relationships between dependents and the head or to resolve potential ambiguities, increasing the number of regressions despite faster overall processing as suggested by shorter TFTs, for instance.

Notably, individual differences in the effect of number of left dependents were most stable—but still only moderately stable at best—in first‐pass measures such as FPRT. This supports the conclusion that, while during early stages of processing, individuals may show stable idiosyncratic gaze behavior to handle complex sentences, during later stages, reading patterns or strategies (e.g., whether to regress or not) are more variable intraindividually.

In summary, we found a clear contrast between the moderate to strong reliability of individual word length effects across measurement occasions and the effects of all other psycholinguistic predictors, which at best exhibited moderate reliability across measurement occasions when assessed with specific ET measures, mostly global measures that include second‐pass readings. As laid out at the beginning of the Discussion, a word's length can induce effects related to basal stages of visual processing, but also related to phonological and morphological demands (e.g., more syllables or greater morphological complexity) as opposed to effects of dependency distance and surprisal that relate to higher‐level syntactic and semantic integration of the current word. Our results suggest that such higher‐level processes are more prone to day‐to‐day inconsistencies in the context of dependency resolution.

#### 5.1.4 Individual differences show moderate reliability between ET measures and SPR times

A key factor influencing cross‐method reliability in our data is the mismatch between the spillover‐sensitive nature of SPR times and the more local timing captured by ET measures. When using a model that is able to account for this difference—comparing spillover‐adjusted SPR to local ET measures—reliability estimates increased substantially. This finding underscores the importance of matching the temporal scope of measures when comparing across methods: ignoring such differences can lead to underestimated correlations and misleading conclusions about the consistency of individual differences.

Overall, compared to measurement reliability across experimental sessions, we observed lower cross‐method reliability across all measurement pairs (i.e., TFT and SPR, FPRT and SPR, RPD and SPR). Interestingly, while across experimental sessions, the random intercept's reliability was by far the most stable by‐subject random effect, across methods, the correlation coefficients for the random intercept are lower than the ones for the word length effect and are of similar magnitude to those observed for lexical frequency and surprisal. The random intercepts obtained with SPR exhibited the highest correlation when compared to the two global ET measures TFT and RPD. This is to be expected since SPR reaction times reflect the cumulative processing effort, which is more aligned with the global than the first‐pass ET measures. As a result, in our study, participants with above‐average random intercepts in global ET measures also tend to have longer above‐average reading times in SPR.

Turning to surprisal, the posterior correlation coefficients between the individual‐level local surprisal effect in ET and the corresponding individual‐level spillover surprisal effect in SPR are higher compared to the reliability estimates of individual surprisal effects within methods (i.e., across measurement occasions), but the individual‐level effects can still only be considered moderately reliable. We hypothesize that the aggregation of more data across two ET and two SPR sessions provides a more stable estimate of individual surprisal effects by reducing potential session‐related trial‐by‐trial variability. This is indeed supported by the higher SNR ratio $\hat{\gamma }$ for the individual surprisal effect estimated in the cross‐method setting (see Table 8). This suggests that, despite the temporal misalignment, the underlying individual differences in sensitivity to surprisal are robust enough to partially persist across paradigms.

The individual lexical frequency effect showed moderate degrees of cross‐method reliability. Compared to the measurement reliability across measurement occasions, the cross‐method reliability of the individual lexical frequency effect is lower, suggesting that this individual effect is not as transferrable across methods as it is across sessions. This may be due to the fact that lexical frequency effects are more associated with early perceptual and oculomotor processes, which differ substantially between the ET and the SPR paradigm. For example, frequency effects are typically strongest in first‐pass measures (Reingold, Yang, & Rayner, 2010)—and have been shown to also be most stable in first‐pass measures across measurement occasions in this work—and are also sensitive to parafoveal preview (Veldre & Andrews, 2016), which is prevented in SPR due to its serial presentation of words. For these reasons, the individual lexical frequency effect may not generalize as well across tasks that differ in their perceptual and motor constraints.

Finally, the individual effects in response to the predictors of syntactic integration—dependency distance and the number of left dependents—showed poor cross‐method reliability. We hypothesize that the main factor contributing to this result is, again, the reading paradigm which shapes how readers resolve syntactically challenging structures; although our SPR incremental‐window paradigm allows for regressions, the behavioral manifestations of syntactic difficulty such as extended re‐reading or regressions may be more fully expressed in ET than in SPR (Ferreira & Henderson, 1990; Just et al., 1982). Moreover, it is also possible that the poor reliability estimate results from the low ratio between true between‐subjects variability of the individual effect and trial‐by‐trial variability, which leads to an attenuation of the correlation coefficient (Rouder et al., 2023).

In summary, individual differences in most of the psycholinguistic effects we examined do not exhibit high cross‐method reliability between ET and the cumulative moving‐window SPR that we deployed. The cumulative window is a less common SPR paradigm relative to noncumulative paradigms (Rayner, 1998), and although it allows for regressions and might appear more naturalistic, it likely encourages reading strategies that diverge from typical reading behavior. Moreover, this paradigm does not reflect processing difficulty on a word‐by‐word, incremental basis: because participants can re‐read earlier material, the resulting reading times conflate initial and later processing efforts (Ferreira & Henderson, 1990; Just et al., 1982). Spillover effects further obscure measurement, as processing difficulty may influence not only the target word but also subsequent words (Boyce & Levy, 2023; Smith & Levy, 2013; Witzel et al., 2012). This makes it difficult to localize the source of processing difficulty with precision. Future work should investigate whether individual‐level effects observed in noncumulative SPR paradigms align better with individual‐level effects in ET measures.

### 5.2 Revisiting the reliability paradox

#### 5.2.1 Mitigating the reliability paradox in naturalistic reading

**Fig. 7:**
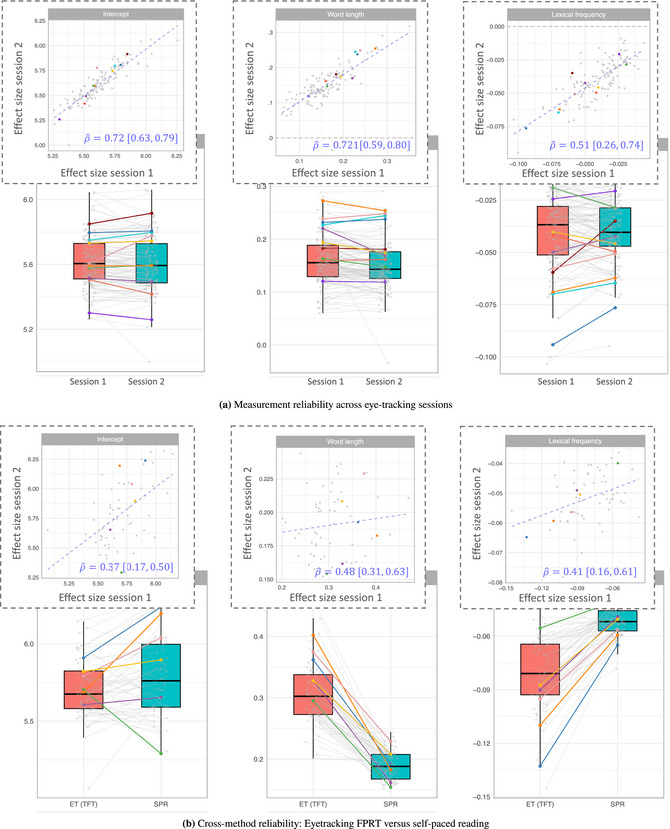
Measurement reliability across measurement occasions (top) and experimental methods (bottom) for total‐fixation times: Each pair of dots represents the estimated effect sizes across two sessions for a specific individual. Ten randomly selected participants are highlighted with different colors. In the top panels, we show the reliability score, quantified by $\hat{\rho }$.

In Fig. 7, we provide a broader perspective on the reliability paradox in naturalistic reading, revisiting it with empirical results from the measurement reliability estimates across measurement occasions and methods. We use TFT as an exemplary ET measure since it aligns most closely with SPR times in the cross‐method comparisons.

Fig. 7a illustrates how, despite significant population‐level effects (low between‐subjects variance) observed across all psycholinguistic predictors (see Section 4.1.1), individual intercepts, word length effects and, to some degree, effects of dependency distance remain consistent across sessions. This result goes against predictions derived from the reliability paradox. We attribute these findings to the relatively high number of measurements from each subject (i.e., the number of words read by each subject) per session (approximately 2000), reducing trial‐by‐trial variance (i.e., $\sigma_{\varepsilon_{x}}^{2}$ in Eq. 4) and helping preserve consistent rankings across participants. This is reminiscent of our second simulation presented in Fig. 2.^20^

However, why is reliability across sessions lower for effects in response to predictors such as lexical frequency, surprisal, and the number of left dependents? According to Eq. A.2 in Online appendix A, the estimated correlation coefficient $\hat{\rho }$ is given by $\rho /(1+\gamma^{2}/M)$, where $\gamma^{2}$ is the SNR variance ratio, $\sigma_{x}^{2}/\sigma_{\varepsilon }^{2}$ (Rouder & Mehrvarz, 2024). Lower reliability for these effects may arise from a low SNR variance ratio ($\gamma^{2}$), either due to high noise variance $\sigma_{\varepsilon }^{2}$ or very low between‐subjects variance $\sigma_{x}^{2}$. Table 8 confirms indeed that between‐subjects variance is low for these predictors, which, according to the reliability formula, directly limits the correlation coefficients.

To further contextualize our observed $\hat{\gamma }$‐ratios, it is helpful to compare them to the values reported by Rouder et al. (2023), who analyzed 24 inhibition tasks and found that trial‐by‐trial variability was typically about eight times greater than the estimated between‐subjects variability (i.e., $\hat{\gamma }$‐values of 0.05–0.36). While our $\hat{\gamma }$‐values span a comparable numerical range (typically between 0.01 and 0.49), it is crucial to note that our model operates on the log‐transformed scale. As a result, $\hat{\gamma }$ quantifies multiplicative differences in reading times across individuals and trials, whereas Rouder et al.'s (2023) ratios reflect additive differences. This distinction implies that these values cannot be compared directly. Still, the qualitative pattern is consistent: in most measures and sessions, trial‐to‐trial variability dominates, with between‐subjects variability accounting for only a small fraction of the total variance. Therefore, although hierarchical models are useful to disentangle different sources of variance, they still suffer from rather poor localization of correlations (Rouder et al., 2023). As indicated by the relatively high SNR ratios for many of the investigated effects, this also holds in the case of modeling reading times in a naturalistic reading paradigm. However, there is one additional potential reason for low reliability estimates, namely, that the individual‐level differences themselves may simply not be reliable. If the true correlation is inherently low, no amount of increased data per individual will substantially improve the estimate. Still, despite the considerable attenuation suggested by our $\hat{\gamma }$‐values, several predictors exhibit moderate to high correlation coefficients. This indicates that at least some psycholinguistic effects give rise to reliable individual differences, even under substantial noise.

#### 5.2.2 Cross‐method reliability: Future directions and challenges

To the best of our knowledge, we have provided the first assessment of cross‐method reliability, comparing ET with cumulative SPR. Future research could investigate the transferability of effects across additional methods such as noncumulative SPR (Just & Carpenter, 1992), maze tasks (Forster, Guerrera, & Elliot, 2009), or Mouse Tracking for Reading (Wilcox et al., 2024). Furthermore, in its current form, our cross‐method approach is only applicable to response variables of the same type (e.g., SPR times vs. continuous ET reading measures). It would be desirable to generalize this framework to enable the comparison of different types of response variables. However, when extending cross‐method reliability assessments, it is important to consider a methodological challenge that arises in this setting: For all individual‐level effects except for surprisal, cross‐method reliability has shown to be lower compared to reliability across measurement occasions within the same method. While all the factors that have been discussed in the previous section for the test‐retest scenario apply here as well, Fig. 7b highlights an additional challenge in measuring the reliability of individual differences.

When examining Fig. 7b, we notice a shift in both the mean as well as the variance of the individual effects, which is particularly pronounced for the individual word length and lexical frequency effects. This can also be confirmed in Table 8, where we note that the standard deviation of the individual effects is consistently smaller in SPR compared to ET. However, the equation for the estimated correlation coefficient $\hat{\rho }=\rho /\left(1+\gamma /M\right)$ is based on several assumptions such as $\mu_{x_{u}}=\mu_{x_{v}}=\mu_{x}$ and $\sigma_{x_{u}}=\sigma_{x_{v}}=\sigma_{x}$, which imply that the true effects and variances in ET and SPR should be identical. According to Eq. A.3 in Online appendix A, differences in effect sizes do not impact reliability, but discrepancies in between‐subjects variances between ET and SPR do. Eq. A.2 only holds when $\sigma_{x_{u}}^{2}=\sigma_{x_{v}}^{2}$, allowing for the maximum reliability estimates with a fixed number of words (M). If the variances diverge, the estimated reliability decreases and deviates further from the true reliability. Thus, significant differences in variability pose a major challenge for accurately estimating the correlation between the two methods. Even with a strong underlying true correlation and a large number of words read per session, approaching this true correlational reliability proves to be very difficult (see Online appendix A).

### 5.3 Methodological considerations

#### 5.3.1 Alternative distribution choices

In this study, we chose to model reading times using a log‐normal distribution, a standard choice in psycholinguistic research due to its ability to account for the right‐skewed nature of reaction times. The log‐normal distribution does not require any additional shape parameters as opposed to potential alternatives and, therefore, reduces the risk of overparameterization, in particular considering the already large number of predictors in our model. Nonetheless, several alternative distributions could be considered for modeling reading times, each with different assumptions about the underlying processes.

The ex‐Gaussian distribution (Hohle, 1965), for instance, assumes a mixture of two components: a normal distribution capturing central tendency and an exponential distribution modeling the long tail of slow responses. This approach is particularly useful for capturing occasional lapses in attention or response inhibition, which result in unusually long reaction times which are not expected in naturalistic reading experiments.

Given that reaction times, including reading times, are often shifted (e.g., there is a lower‐bound, resulting from motor execution or stimulus encoding), additional shift parameters can be introduced in order to account for this fact, resulting in the shifted log‐normal distribution (Rouder, 2005). Although the shift parameter might result in a slightly better model fit, it requires the estimation of an additional parameter in an already complex model. Furthermore, we do not expect this to affect our reliability estimates as the shift is likely absorbed by the global intercept.

Lastly, there is an additional set of distributions that would be suitable to analyze reading times, but specifically model the time until an event (e.g., a decision or neuronal spike) occurs, such as the shifted Weibull distribution (Onuoha, Osuji, Etaga, & Obulezi, 2023), or the Wald (or inverse Gaussian) distribution (Lo & Andrews, 2015). Although they may be useful for psycholinguistic experiments such as lexical decision or visual search tasks, it does not pose a natural choice for naturalistic reading, where each word constitutes its own trial. While we chose the log‐normal model based on its interpretability and fit to our data, future work could systematically compare these alternative models.

#### 5.3.2 Toward theoretically grounded models for the assessment of measurement reliability

While our current analyses provide a detailed statistical account of measurement reliability, they remain agnostic about the cognitive processes that generate the observed data. As Haines, Sullivan‐Toole, and Olino (2023) argue, traditional statistical approaches often fail to capture individual differences in cognitive tasks because they do not adequately reflect the underlying data‐generating mechanisms. Similarly, Xu and Stocco (2021) emphasize the need for computational models that explicitly incorporate cognitive and psychological mechanisms, as such models can reveal individual variability that conventional summary statistics obscure. Although we chose the psycholinguistic predictors such that the model incorporates the most important sources of variance reported in the sentence processing literature, it does not make direct assumptions about how reading times are generated as opposed to models of eye movements such as SEAM (Rabe, Paape, Mertzen, Vasishth, & Engbert, 2024) or Über‐reader (Veldre, Yu, Andrews, & Reichle, 2020). Future work could explore how generative modeling techniques might bridge the gap between different experimental paradigms, enabling a principled comparison of response variables across methods. By grounding measurement reliability in cognitive theory, this approach could enhance both the interpretability and the robustness of cross‐method findings.

### 5.4 Implications for theories of sentence processing

The fact that many predictors only exhibited robust measurement reliability when assessed with a specific method and measure suggests that, when studying individual differences in sentence processing, one should (i) choose the method in which the particular predictor has been shown to be reliable across experimental sessions, and (ii) keep in mind that measurement reliability estimates might still not be transferable across different methods and need to be assessed separately.

Our approach uses experimentally nonmanipulated texts in a naturalistic reading setting, possibly resulting in less pronounced population‐level effects than in traditional minimal‐pair experiments. Targeted minimal‐pair designs (e.g., high‐ vs. low‐surprisal conditions) might elicit larger population‐level effects. How the potentially stronger effects at the population level will affect the between‐sessions reliability of individual‐level effects in minimal‐pair experiments remains an open question to be addressed in future research. However, due to the impact of the reliability paradox, under a low trial number scenario, the low individual reliability of the effects we investigated is expected to persist in targeted minimal‐pair designs. This is because in sentence processing research, in minimal‐pair experiments, typically, one sentence (rather than one word) is treated as one trial, which, in turn, makes it much more costly to increase trial numbers.

Our findings have major implications for theories of sentence processing at large. On the one hand, when investigating and modeling the variability in human reading behavior and developing theories that generalize to the individual level, we should be cautious about incorporating phenomena that have been shown to be replicable at the population level (Kidd et al., 2018) unless shown to be measurement reliable on the individual level too. By contrast, we should rather start considering phenomena that have not been well‐established at the population level. These phenomena may include, for instance, interference effects in reflexives for which to this date, studies report contradictory and inconclusive results (Dillon, Mishler, Sloggett, & Phillips, 2013; Jäger, Mertzen, Van Dyke, & Vasishth, 2020; Laurinavichyute, Jäger, Akinina, Roß, & Dragoy, 2017), or characteristic scanpath signatures that reflect stable individual reading strategies (von der Malsburg, & Vasishth, 2011), as well as other general eye movement patterns that capture idiosyncratic aspects of reading behavior. Finally, we also suggest considering individual task demands that require readers to adjust their behavior in order to cope with a task at hand. Once a set of measurement reliable phenomena has been established, theories of sentence processing need to be modulated to account for individual differences. On the other hand, a large body of correlational studies investigating the associations between individual effects and cognitive measures, in the spirit of Kuperman & Van Dyke (2011), Cunnings and Felser (2013), Nicenboim et al. (2015), or Vuong and Martin (2014), would need to be revisited: if individual effects are not measurement reliable, we cannot rule out that suggested correlations with specific individual characteristics, such as high working memory capacity or low cognitive control, are not reproducible. Future research could investigate the extent to which the reliability of psycholinguistic effects is associated with participants' cognitive capacities, current state of mind (e.g., fatigue), or demographic factors such as age.

## 6 Conclusion

In this work, we collected German naturalistic reading data from 136 participants using two widely applied psycholinguistic methods: ET and SPR. One hundred and thirty‐one participants completed two separate ET sessions, and 65 of them also participated in two additional SPR sessions, resulting in a total of 392 experimental sessions. Complementarily, we conducted comprehensive psychometric tests to evaluate the cognitive capacities of the 65 participants who participated in all four sessions. We release this data set as the InDiCo. We deployed two‐task Bayesian hierarchical models to assess the measurement reliability between experimental sessions and across methods of five key psycholinguistic predictors: word length, lexical frequency, surprisal, dependency distance, and number of left dependents.

Our results indicate that measurement reliability of overall individual reading patterns and individual word‐length effects is relatively strong across experimental sessions, and moderate across methods (i.e., between ET and cumulative SPR). Moreover, for most psycholinguistic predictors, measurement reliability across experimental sessions is stronger in global reading measures than in first‐pass measures.

Based on our results, when studying individual differences in sentence processing, we recommend researchers to (i) choose reading measures that demonstrate high individual‐level reliability for the predictor of interest, and (ii) consider the transferability of effects across methods.

In conclusion, this study presents the first detailed investigation of measurement reliability for individual differences in naturalistic reading across four measurement occasions and two experimental methods. In addition, it is the first such study conducted in German, focusing on five key psycholinguistic effects that psycholinguistic theories of sentence processing build on. Our results challenge previous research on individual differences in sentence processing that focused phenomena that are well‐established on the group‐level, and underscore the need to explore new phenomena that may uncover reliable individual differences. This is crucial for developing robust, individualized theories of sentence processing that better reflect the diversity of reading behavior.

## Supporting information

Online Appendix

## Notes

1
https://github.com/DiLi‐Lab/measurement‐reliability.

2 In this simulation, we assume that all items capture the effect of interest equally well, such that the only source of *trial‐by‐trial variability* is the measurement error (i.e., the residual noise added to the true score). For simplicity, we use the term measurement error here. Later, in Section 4.3, we adopt the broader term trial‐by‐trial variability, following Rouder, Kumar, and Haaf (2023), to denote an umbrella category that covers any unmodeled sources of variance, such as systematic differences across items.

3 Note, however, that for effects caused by relative clause structures, naturalistic reading has particularly few trials compared to a minimal‐pair experiment. Moreover, oftentimes, the variance of the predictor variable (e.g., surprisal) is lower in natural texts than in a planned experiment with minimal‐pair stimuli.

4 Note that while split‐half reliability is applicable in minimal‐pair sentence‐reading studies such as Staub (2021), this is not the case for naturalistic reading paradigms that use long, cohesive texts (e.g., four texts per session, as in our study), where each word constitutes a single item. The cohesive nature of the texts makes it impossible to split the texts in a meaningful way.

5 To validate this assumption, we present a confirmatory factor analysis (CFA) in Online appendix B.

6 Note that we specifically use the terms *first‐pass* and *later‐pass* to avoid confusion with the terms *early* and *late*, which are used in models of sentence processing and make explicit assumptions about the order of *processes* during comprehension (Clifton, Staub, & Rayner, 2007; Frazier, 1987). In this work, we adopt the theory‐agnostic terms *first‐pass*, *later‐pass*, and *global*, which refer solely to the timing of the reading measure.

7 The adverse conditions were (i) sleep‐deprivation and (ii) the influence of small amounts of alcohol. These sessions served as a pilot for further experiments in a driving simulator and are unrelated to the present study.

8 We only consider right dependents—that is, dependents whose head has been encountered already.

9 The dLex data base can be accessed via http://www.dlexdb.de.

10
https://huggingface.co/malteos/gpt2‐xl‐wechsel‐german.

11
https://spacy.io/models/de#de_dep_news_trf.

12 We used the Python‐based implementation provided by Krakowczyk et al. (2023): https://github.com/aeye‐lab/pymovements.

13 Note that for our study, a word was considered skipped if no fixations have been recorded on it. In some studies, the term “skip” is used to refer to *first‐pass* skips.

14 Note that we enumerate the words along all texts to avoid an additional index denoting the different texts.

15 Note that for the zero‐inflated Poisson model, the proportion of excess zeros is independent of the psycholinguistic predictors. While this posits a limit to our model, this simplification was necessary in order to ensure model convergence.

16 Note that no by‐item random effects were included. Since in naturalistic reading, in theory, each token constitutes its own item, we do not have enough data to estimate such additional random effects. Given that the model's random‐effects structure is already very complex, we also refrained from fitting additional by‐text or by‐screen random effects.

17 Although we can assume similar spillover effects between both sessions, we provide the reliability estimates based on the spillover‐model defined in Eq. 6 in Appendix D.1.

18 Here, following Rouder et al. (2023), we use the term *trial‐by‐trial variability* to denote an umbrella term that covers any unmodeled sources of variance at the trial level. Because we did not include random intercepts or slopes for items, this variability may also reflect systematic differences between items, in addition to measurement error. Including item‐level random effects would overparametrize our model relative to the available data.

19 Note that for count‐based response variables, as well as binary response variables, no dispersion parameter is estimated; therefore, we cannot calculate the SNR ratio.

20 Note that in our study, words are analogous to trials in Fig. 2.
