# Supplementary material for: Replicate Me if You Can: Assessing Measurement Reliability of Individual Differences in Reading Across Measurement Occasions and Methods
Source: Cogn Sci. 2025 Dec 30;50(1):e70121. doi: 10.1111/cogs.70121 (PMC12752730; doi:10.1111/cogs.70121)
Supplement: Supplementary file 1 — Online Appendix [file COGS-50-e70121-s001.pdf]

APPENDIX

A THE RELIABILITY PARADOX AND HOW TO BYPASS IT IN NATURALISTIC READING

Suppose we have two sets of effect size samples, $x_{u1}, x_{u2}, \ldots, x_{un}$ and $x_{v1}, x_{v2}, \ldots, x_{vn}$, from N participants across two sessions, u and v. These effects are based on m repeated measures (corresponding to M words read in both sessions), each measured with error $\varepsilon \sim N(\mu_\varepsilon, \sigma_\varepsilon^2)$ and the effects also follow a bivariate normal distribution with true correlation ρ:

$$\mathbf{x} \sim \mathcal{N}_2\left(\begin{pmatrix}\mu_{x_u}\\ \mu_{x_v}\end{pmatrix}, \begin{pmatrix}\sigma_{x_u}^2 & \rho\sigma_{x_u}\sigma_{x_v}\\ \rho\sigma_{x_u}\sigma_{x_v} & \sigma_{x_v}^2\end{pmatrix}\right),$$

where we assume that $\mu_{x_u} = \mu_{x_v} = \mu_x$ and $\sigma_{x_u} = \sigma_{x_v} = \sigma_x$.
The measurements in session u gives in total $n \times m$ values, $u_{11}, u_{12}, ..., u_{ij}, ...u_{nm}$, with $u_{ij} = x_{ui} + \varepsilon_{uij}$ being the measured effect of the i-th participant on the j-th word, where ε_{uij} denotes the corresponding measurement error. For the i-th participant, the average effect over m words is $\bar{u}_i = x_{ui} + \bar{\varepsilon}_{ui}$. Further averaging over all the n participants gives the overall average effect in this session $\bar{\bar{u}} = \bar{x}_u + \bar{\bar{\varepsilon}}_u$. The observational between-subjects variance over all participants and all words is $s_u^2 = s_{x_u}^2 + s_{\bar{\varepsilon}_u}^2$, where $s_{\bar{\varepsilon}_u}$ is just the standard error of the mean of ε_{uij}, which, as an excellent approximation, is expressed as $s_{\bar{\varepsilon}_u} \approx \frac{\sigma_\varepsilon}{\sqrt{M}}$. Similarly, the average effect and variance in session v is $\bar{\bar{v}} = \bar{x}_v + \bar{\bar{\varepsilon}}_v$ and $s_v^2 = s_{x_v}^2 + \sigma_\varepsilon^2/M$, respectively.
The correlation between measurements of all the participants in session u and v can be written

$$\begin{aligned}\hat{\rho}_{uv} &= \frac{E[(\bar{u}_i - \bar{\bar{u}})(\bar{v}_i - \bar{\bar{v}})]}{s_u s_v}\\ &= \frac{\frac{1}{n}\sum_{i=1}^{n}[(x_{ui} + \bar{\varepsilon}_{ui}) - (\bar{x}_u + \bar{\bar{\varepsilon}}_u)][(x_{vi} + \bar{\varepsilon}_{vi}) - (\bar{x}_v + \bar{\bar{\varepsilon}}_v)]}{\sqrt{s_{x_u}^2 + \sigma_\varepsilon^2/M}\sqrt{s_{x_v}^2 + \sigma_\varepsilon^2/M}}.\end{aligned} \tag{A1}$$

Suppose the number n is sufficient so that the between-subjects variance in the two sessions is well estimated, $s_{x_v}^2 \approx s_{x_v}^2 \approx \sigma_x^2$, and the measurement errors in the two sessions are independent from each other and are also independent from $\mathbf{x}$, then:

$$\begin{aligned}\hat{\rho}_{uv} &\approx \frac{\frac{1}{n}\sum_{i=1}^{n}(x_{ui} - \bar{x}_u)(x_{vi} - \bar{x}_v)}{\sigma_x^2 + \sigma_\varepsilon^2/M}\\ &= \frac{\rho\sigma_x^2}{\sigma_x^2 + \sigma_\varepsilon^2/M}\\ &= \frac{\rho}{1 + \gamma^2/M},\end{aligned} \tag{A2}$$

where γ^2 is defined as $\gamma^2 \equiv \sigma_\varepsilon^2/\sigma_x^2$. This γ^2 is the inverse of the signal-to-noise variance ratio proposed by Rouder and Mehrvarz (2024). As noted by Rouder and Mehrvarz (2024), γ^2 is an invariant measure that governs the rate of increase of the correlation estimate as the number of trials (or words in naturalistic reading) increases. Our correlation formula is similar to that in Rouder and Mehrvarz (2024), but with distinctions: we differentiate between the true underlying correlation and the estimated correlation, and we deal with continuous variables in naturalistic reading. The absence of coefficient 2 in our formula is due to this continuous variable setting, whereas the coefficient in Rouder and Mehrvarz (2024) comes from contrast coding for binary variables.
Thus, by increasing the number of words m read in each session, the correlation estimate ρ_{uv} improves and gradually approaches the true underlying correlation, even if the between-subjects variance σ_x^2 is very small and the measurement noise is large, making the reliability paradox less of a problem for studying individual differences.
However, this optimistic view relies on several assumptions that may not hold in real-world scenarios. For instance, in our cross-method reliability experiment, the assumptions $\mu_{x_u} = \mu_{x_v} = \mu_x$ and $\sigma_{x_u} = \sigma_{x_v} = \sigma_x$ are easily violated. What impact would this have on the estimated correlations?
The formula for $\hat{\rho}_{uv}$ shows that the means μ_{x_u} and μ_{x_v} do not influence the estimate, so differences between them are irrelevant. However, if $\sigma_{x_u} \neq \sigma_{x_v}$, we can no longer represent $\hat{\rho}_{uv}$ as $\rho\sigma_x^2/(\sigma_x^2 + \sigma_\varepsilon^2/M)$. Instead, we have:

$$
\begin{aligned}
\hat{\rho}_{uv} &\approx \frac{\frac{1}{n}\sum_{i=1}^{N}(x_{ui}-\bar{x}_u)(x_{vi}-\bar{x}_v)}{\sqrt{(\sigma^2_{x_u}+\sigma^2_{\varepsilon}/M)(\sigma^2_{x_v}+\sigma^2_{\varepsilon}/M)}} \\
&= \frac{\rho\sigma_{x_u}\sigma_{x_v}}{\sqrt{(\sigma^2_{x_u}+\sigma^2_{\varepsilon}/M)(\sigma^2_{x_v}+\sigma^2_{\varepsilon}/M)}} \\
&= \frac{\rho\sigma_{x_u}\sigma_{x_v}}{\sqrt{\sigma^2_{x_u}\sigma^2_{x_v}+\sigma^2_{\varepsilon}(\sigma^2_{x_u}+\sigma^2_{x_v})/M+\sigma^4_{\varepsilon}/M^2}} \\
&\leq \frac{\rho}{\sqrt{1+(\sigma^2_{\varepsilon}/M)\cdot(2\sigma_{x_u}\sigma_{x_v}/\sigma^2_{x_u}\sigma^2_{x_v})+\sigma^4_{\varepsilon}/(M^2\sigma^2_{x_u}\sigma^2_{x_v})}} \\
&\overset{\max}{=} \frac{\rho}{\sqrt{1+(\sigma^2_{\varepsilon}/M)\cdot(2/\sigma^2_{x})+\sigma^4_{\varepsilon}/(M^2\sigma^4_{x})}} \\
&= \frac{\rho}{1+\sigma^2_{\varepsilon}/(M\sigma^2_{x})} \\
&= \frac{\rho}{1+\gamma^2/M}.
\end{aligned}
\tag{A3}
$$

This implies that when $\sigma_{x_u} = \sigma_{x_v}$, the estimated correlation is maximized. As σ_{x_u} and σ_{x_v} diverge, the estimated correlation deviates further away from the true underlying correlation.

B CONFIRMATORY FACTOR ANALYSIS

We conduct a confirmatory factor analysis (CFA) in order to assess the degree to which the different texts presented across four sessions (16 in total) assess the same single underlying psychological construct of reading comprehension. This allows us to assess to what extent our different texts consistently measure the same underlying construct, which is important for interpreting our experimental setting in a repeated-measures context, more specifically to what degree our experimental setting can be considered a *test-retest* setting.

We use one-factor-CFA model to test hypothesis that the covariance in responses (reading times and comprehension question accuracy) among texts is due to a single common latent factor, i.e., *reading comprehension ability*. Formally, the model is defined as follows:

$$
y_{ti} = \lambda_t \xi_i + u_{ti}, \tag{B4}
$$

where y_{ti} is individual i's observed response, e.g., an individual's average reading measure or average comprehension question accuracy on text t. ξ_i is individual i's score on the latent common factor, u_{ti} is individual i's score on the tth latent unique factor, and λ_t is the factor loading that indicates the relation between the text t and the latent common factor.

We perform the CFA on four different response variables: first-pass reading times (eye-tracking), total fixation times (eye-tracking), word reading times (self-paced reading) and response accuracy (comprehension questions). Due to the counter-balancing as well as the fact that each individual reads only 8 out of the 16 texts in eye-tracking or self-paced reading, respectively, we had to fit separate models on subsets of texts for which the same data type (e.g., ET) of multiple participants was available (e.g., only 25 participants were exposed to texts 9, 10, 15 and 16 during ET). We chose the four mutually exclusive sets of text combinations for which most data was available (see column Text IDs in Table B1).

We fit the models using the R-package `lavaan` and provide several fit indices for model evaluation:

The chi-squared test assesses the difference between the observed and expected covariance matrices, with values closer to zero indicating a better fit.

The Comparative Fit Index (CFI; see Eq. B5 adjusts for sample size issues inherent in the chi-squared test and compares the proposed model with a null model assuming that the observed measures do not share a common latent factor. Values of CFI above 0.95 indicate an excellent fit, while values between 0.90 and 0.95 suggest a good fit.

$$CFI = 1 - \frac{\max\left[\chi^2_{\text{proposed model}} - df_{\text{proposed model}}, 0\right]}{\max\left[\chi^2_{\text{null model}} - df_{\text{null model}}, 0\right]} \tag{B5}$$

Similarly, the Tucker Lewis Index (TLI or NNFI; cf Eq. B6) also combines parsimony with a comparative index, with values above 0.95 indicating a very good fit.

$$\text{TLI} = \frac{\frac{\chi^2_{\text{null model}}}{\text{df}_{\text{null model}}} - \frac{\chi^2_{\text{proposed model}}}{\text{df}_{\text{proposed model}}}}{\frac{\chi^2_{\text{null model}}}{\text{df}_{\text{null model}}} - 1} \tag{B6}$$

The results of the CFA are presented in Table B1. Bold numbers indicate good model fit, i.e., support the hypothesis that the observed variables load onto a single latent factor representing reading comprehension ability. The CFI in particular suggests adequate model fit of the eye movement measures with all values ≥ 0.90. Regarding self-paced reading times, only the CFI for set (3,4,13,14) indicates adequate model fit. For text set (1,2,7,8), the CFI is 0.70, suggesting that readers interact differently with these texts during self-paced reading.

Measure	n_{obs}	Text IDs	Chi-square test	CFI	TLI
ET(FPRT)	49	3,4,13,14	$\chi^2(2) = 8.10, p < 0.05$	**0.98**	**0.95**
ET(FPRT)	43	1,2,5,6	$\chi^2(2) = 8.23, p < 0.05$	**0.97**	**0.92**
ET(FPRT)	25	9,10,15,16	$\chi^2(2) = 14.86, p < 0.001$	**0.90**	0.70
ET(FPRT)	27	7,8,11,12	$\chi^2(2) = 14.73, p < 0.001$	**0.94**	0.81
ET(TFT)	49	3,4,13,14	$\chi^2(2) = 0.69, p = 0.709$	**1.00**	**1.01**
ET(TFT)	43	1,2,5,6	$\chi^2(2) = 5.69, p = 0.058$	**0.98**	**0.95**
ET(TFT)	25	9,10,15,16	$\chi^2(2) = 7.13, p < 0.05$	**0.96**	0.88
ET(TFT)	27	7,8,11,12	$\chi^2(2) = 12.27, p < 0.01$	**0.93**	0.80
SPR	17	1,2,7,8	$\chi^2(2) = 25.04, p < 0.001$	0.70	0.11
SPR	37	3,4,13,14	$\chi^2(2) = 5.33, p = 0.07$	**0.98**	**0.95**
SPR	13	5,6,9,10	$\chi^2(2) = 9.86, p < 0.01$	0.86	0.58
SPR	11	11,12,15,16	$\chi^2(2) = 12.92, p < 0.01$	0.87	0.61
Accuracy	98	All	$\chi^2(104) = 126.63, p = 0.065$	**0.92**	**0.91**

TABLE B1 CFA results with mean reading measures (FPRT, TFT, SPR) and comprehension question accuracy on a set of texts as observed indicators for a common single latent factor of *reading comprehension*. n_{obs} refers to the number of individuals that were exposed to the same set of texts. The Chi-square test, Comparative Fit Index (CFI) and Tucker Lewis Index (TLI) indicate the goodness-of-fit assuming a common factor for a given set of texts.

C PSYCHOMETRIC ASSESSMENTS

C.1 Assessment of cognitive control

C.1.1 Verbal cognitive control

Verbal cognitive control was assessed using a German adaptation[1] of the *Stroop task* (Stroop 1992). Participants saw a color adjective (BLAU, ROT, or GELB) or the string %%%%, displayed in blue, red, or yellow fonts. They were instructed to identify the ink color as quickly as possible by pressing the corresponding key. Finger positioning was standardized for optimal response speed.[2] Each stimulus appeared in the center of the screen after a 350 ms fixation cross. Stimuli, shown in uppercase Courier

[1] The code for our German adaptations of the Stroop and Simon tasks is available here: `https://github.com/DiLi-Lab/stroop-simon-german`.

[2] In the original Stroop task, green was also included. However, we excluded it in the German version because "yellow" and "green" both start with "G" in German, and the inclusion of both red and green could pose challenges for color-blind participants.

New font on a light grey background, were classified into three conditions: neutral (%%%% displayed in one of the three colors), congruent (color word and ink match), and incongruent (color word and ink mismatch). Participants completed 12 practice trials with feedback, followed by 72 trials (24 trials per condition) without feedback. They were encouraged to respond quickly and accurately. The reaction times are calculated by subtracting the reaction times of the incongruent condition from those of the congruent condition. Accuracy is defined as the number of correct responses across all conditions per participant.

C.1.2 Non-verbal cognitive control

The *Simon task*, a non-verbal equivalent of the Stroop task, was also used. Instead of words, blue or red squares appeared on the left, right, or center of the screen. Participants were instructed to press the "s" key with their left hand when a blue square appeared and the "k" key with their right hand, when the red square appeared, regardless of the positions of the symbols on the screen. In the congruent condition the symbols appeared on the same side as the key press, in the incongruent condition they appeared on the opposite side. In the neutral condition, they appear in the center of the screen.
The reaction times are calculated by subtracting the reaction times of the incongruent condition from those of the congruent condition. Accuracy is defined as the number of correct responses across all conditions per participant.
The paper-based *Frankfurter Aufmerksamkeits-Inventar 2* (FAIR-2, Moosbrugger, Oehlschlägel, & Steinwascher 2011) offers a detailed assessment of non-verbal cognitive control, measuring attentional performance, attention quality, and attention continuity. Participants received instructions on paper and completed a practice trial to familiarize themselves with the task. In the following test trial, they had three minutes per page to identify target symbols (e.g., circles with two dots) from a page with 16 rows of 20 symbols. The task, including instructions, took about 10 minutes to complete.
The *attention performance* measures the number of correctly marked items minus twice the sum of errors, reflecting executive attention and working speed. The *quality of attention* is derived by dividing the attention performance measure by the total number of items evaluated, taking into account precision and processing speed. The *attention continuity* (K-value) is the product of the attentional performance measure and the quality of attention measure, indicating the individual's trade-off between speed and accuracy, and is often interpreted as a measure of working style. Quantile scores were derived from norm tables.

C.2 Assessment of working memory capacity

We used the working memory capacity (WMC) test battery developed by Lewandowsky, Oberauer, Yang, and Ecker (2010), re-implemented in Python (Krakowczyk 2023)[3]. This battery includes a sentence span task targeting verbal memory and three tasks targeting non-verbal memory: a memory updating task, an operation span task, and a spatial short-term memory task. Each task took approximately 10 minutes to complete, with response times and accuracy recorded.

C.2.1 Verbal working memory capacity

In the *sentence span task*, participants were presented with a sentence and had to judge its meaningfulness. They pressed the left arrow on a keyboard if the sentence was meaningless e.g., "As the tourist watched the dolphin danced a waltz" and the right arrow if it was meaningful e.g., "As the lion attacked the baboon watched from its cage", with a maximum response time of 4 seconds. The number of meaningful and meaningless sentences was the same. After each sentence, a consonant letter appeared on the screen for one second, which participants needed to remember for later serial recall. The task included 15 trials. Sentences and consonants were presented as sets, ranging from four to eight. After the sequence, participants were prompted to recall the consonants in the order of their presentation, typing as many letters as they could remember and guess, if necessary. Accuracy was calculated as the proportion of correctly recalled letters per set and averaged across all trials.

C.2.2 Non-verbal working memory capacity

The *operation span task* was similar to the sentence span task. Instead of reading sentences, participants alternated between solving arithmetic equations and memorizing consonant letters. They were asked to judge the accuracy of the arithmetic equations by pressing the left arrow if the sentence was incorrect and the right arrow if it was correct, with a maximum response time of 3 seconds. After judging each equation, a consonant appeared for one second to be recalled later in the correct sequence. The

[3]The original test battery was implemented in Matlab and is publicly available, though no longer maintained. The Python re-implementation is available at `https://github.com/aeye-lab/python-wmc-battery`.

task included 15 trials, with sets ranging from four to eight equation-consonant pairs. Participants took self-paced breaks every three trials. Accuracy was calculated as the proportion of correctly recalled letters per set and averaged across all trials.

In the *memory updating task*, participants memorized an initial set of digits, each displayed in a separate box on the screen, and updated these digits through arithmetic operations. The set size varied from three to five boxes with digits, with digits presented one by one for one second each. Afterward, arithmetic cues (e.g., "+3" or "−2") appeared in the boxes, one by one, for 1.3 seconds each, prompting participants to update the memorized digit accordingly. Operations ranged from −7 to +7, with interim and final results ranging between 1 and 9. The number of updates varied between two and six, with some boxes updated multiple times and others not at all. Participants then recalled the final digit in each box when prompted, with no time limit for recall and no feedback provided. The task included 15 trials. Accuracy was calculated as the proportion of correct final results of the operations of each box, averaged across all trials.

In the *spatial short-term memory task*, participants were asked to remember the locations of dots in a 10×10 grid, with set sizes ranging from two to six dots per trial. The dots appeared one by one in random grid cells (excluding corners) for 900 ms each. Participants were instructed to focus on the spatial relations between the dots rather than their absolute positions. After all dots were presented, participants reproduced the pattern by clicking on the cells of an empty grid. The order of clicks as well as the absolute position was irrelevant, and there was no time limit; participants could adjust their responses until satisfied. The task consisted of 30 trials, with six trials for each set size. Accuracy was calculated as a similarity measure of the presented and recalled patterns as specified by Lewandowsky et al. (2010).

C.3 Intelligence

C.3.1 Verbal intelligence

We assessed participants' verbal intelligence using the two verbal subtests (1 and 3) from the Reynolds Intellectual Assessment Scales and Screening (RIAS, Hagmann-von Arx & Grob 2014).[4] Subtest 1 focused on vocabulary and verbal reasoning. The experimenter read a question and participants responded with one word (e.g., Q: "What is tall, has a very bright light, and guides ships during the night?" – A: "Lighthouse". There was no time limit, and the task consisted of up to 25 trials. The task was terminated after three consecutive incorrect answers. The sum of the correct responses was recorded.

Subtest 3 assessed analytical verbal logical thinking, requiring participants to complete a sentence read aloud by the experimenter (e.g., Q: "Wick is to candle as bulb is to?" — A: "Lamp" or "Chandelier"). There was no time limit or feedback, and the task consisted of up to 26 trials. The task was terminated after three consecutive incorrect responses and the sum of correct responses was recorded. Each subtest took approximately 10 minutes to complete.

In addition to the verbal RIAS subtests, we administered the paper-based *MWT-B — Mehrfachwahl-Wortschatz-Intelligenztest* (Lehrl 2005) to measure participants' vocabulary size. Each item presented a list of five words, with only one being a real word and the others pseudo-words. Participants were instructed to cross out the real word. The test consisted of 37 items and had no time limit, typically taking about five minutes to complete. Accuracy was defined as the number of correctly crossed-out words.

C.3.2 Non-verbal intelligence

We assessed non-verbal intelligence using RIAS subtests 2 and 4, both targeting non-verbal cognitive abilities.

Subtest 2 measures non-verbal reasoning skills on the basis of categorization abilities, and requires also the use of spatial abilities and visual imagery. Participants identified which picture, out of six, did not fit. The pictures could be objects, letters, or patterns. They had two attempts per item, with a 30-second limit for the first attempt and 20 seconds for the second. Feedback was provided, and the correct answer was explained if both attempts were incorrect. The task included up to 35 trials, with termination after three consecutive incorrect answers. Scores were 2 points for correct answers on the first attempt and 1 point for the second attempt.

Subtest 4 assesses analytical thinking on the basis of visual object displays. Participants identified what was missing from a drawing (e.g., a helicopter's tail rotor). They had two attempts, with 20 seconds for the first and 10 seconds for the second. Feedback was provided for incorrect answers, and the correct answer was explained if both attempts were incorrect. The task included up to 21 trials, with 2 points awarded for correct first attempts and 1 point for correct second attempts. Termination occurred after three consecutive incorrect answers.

[4] We did not administer the subtests specifically targeting working memory capacity since, given the relevance of working memory for psycholinguistic theories, we opted for assessing working memory capacity in a more fine-grained way using the Lewandowsky et al. (2010) test battery described above.

C.4 Reading fluency

To assess reading fluency, we administered the reading section (Form A) of the Lese- und Rechtschreibtest (SLRT-II) (Moll & Landerl 2010). The test includes two parts: word reading which targets *lexical reading fluency*, and pseudo-word reading which targets *non-lexical reading fluency*. In the first part, participants were instructed to read a list of German words aloud as quickly as possible within one minute without making mistakes. The experimenter timed them for one minute and recorded their progress through the list, which consisted of 156 words across 8 rows. The second part followed the same procedure but used pseudo-words instead, also totaling 156 items across 8 rows. Accuracy was computed as the number of read items, minus incorrectly read aloud or missed items.

D ADDITIONAL RESULTS

D.1 Measurement reliability across measurement occasions with spillover effects

To further investigate the influence of spillover effects on measurement reliability across measurement occasions and to provide an additional point of reference for the models incorporating spillover effects that were fitted on cross-method data (see Section 3.2.2), we fitted the "spillover"-model defined in Equation 6 for the cross-method setting on the data recorded using the same method across the two measurement occasions. We report reliability estimates in Table D2.

Measure	Locality	Word length	Lexical freq.	Surprisal	Dependency dis.	Num. left dep.	Intercept
**FFD**	local	**0.53** [0.31, 0.71]	**0.37** [0.09, 0.61]	0.17 [-0.20, 0.51]	0.32 [-0.06, 0.62]	0.31 [-0.01, 0.59]	**0.71** [0.62, 0.78]
	spillover	**0.37** [0.14, 0.59]	**0.34** [0.05, 0.61]	-0.01 [-0.39, 0.37]	0.21 [-0.08, 0.49]	**0.36** [0.05, 0.63]	
**FPReg**	local	**0.48** [0.19, 0.70]	0.22 [-0.16, 0.54]	0.02 [-0.35, 0.38]	0.26 [-0.08, 0.56]	0.10 [-0.26, 0.44]	**0.84** [0.78, 0.89]
	spillover	**0.52** [0.33, 0.69]	0.29 [-0.04, 0.58]	0.06 [-0.33, 0.43]	0.25 [-0.11, 0.57]	0.09 [-0.29, 0.45]	
**FPRT**	local	**0.73** [0.62, 0.82]	**0.55** [0.33, 0.73]	0.11 [-0.27, 0.46]	**0.50** [0.23, 0.72]	**0.40** [0.14, 0.64]	**0.72** [0.63, 0.79]
	spillover	**0.46** [0.27, 0.64]	0.24 [-0.06, 0.52]	0.03 [-0.36, 0.42]	**0.37** [0.11, 0.60]	**0.34** [0.04, 0.61]	
**RPD**	local	**0.74** [0.64, 0.82]	**0.41** [0.17, 0.63]	0.19 [-0.10, 0.46]	**0.57** [0.35, 0.75]	0.12 [-0.22, 0.44]	**0.73** [0.65, 0.80]
	spillover	**0.30** [0.04, 0.54]	**0.29** [0.01, 0.55]	0.06 [-0.33, 0.45]	0.19 [-0.13, 0.50]	0.19 [-0.17, 0.52]	
**SKIP**	local	**0.55** [0.41, 0.66]	**0.33** [0.07, 0.58]	0.06 [-0.30, 0.41]	**0.50** [0.24, 0.72]	**0.33** [0.02, 0.61]	**0.74** [0.67, 0.81]
	spillover	**0.38** [0.18, 0.57]	**0.39** [0.14, 0.62]	0.01 [-0.38, 0.38]	0.17 [-0.19, 0.50]	0.25 [-0.03, 0.51]	
**TFT**	local	**0.67** [0.55, 0.77]	**0.40** [0.16, 0.62]	0.20 [-0.06, 0.46]	**0.59** [0.38, 0.77]	**0.33** [0.02, 0.61]	**0.70** [0.61, 0.78]
	spillover	**0.33** [0.11, 0.52]	0.28 [-0.00, 0.55]	0.04 [-0.33, 0.39]	**0.30** [0.01, 0.56]	0.25 [-0.07, 0.55]	
**SPR**	local	**0.62** [0.43, 0.77]	0.06 [-0.23, 0.36]	0.17 [-0.15, 0.46]	0.28 [-0.04, 0.56]	0.08 [-0.29, 0.43]	**0.69** [0.56, 0.80]
	spillover	**0.43** [0.21, 0.61]	0.21 [-0.11, 0.52]	0.26 [-0.03, 0.52]	**0.38** [0.14, 0.61]	0.13 [-0.24, 0.47]	

TABLE D2 Measurement reliability of local and spillover psycholinguistic effects across measurement occasions: Posterior distributions of the correlation coefficients for measurement reliability across measurement occasions of different psycholinguistic predictors and reading measures from the eye-tracking and self-paced reading (SPR) data. Results are based on the spillover model defined in Equation 6. We present the mean estimates and the 95% credible intervals of both the local and spillover effects. Bold font denotes that 0 is not included in the 95% credible interval.

Since adding spillover effects may also affect both trial-by-trial variability (measured via the dispersion parameter in the models fitted on continuous response variables), we show the ratio $\hat{\gamma}_m$ between the residual standard deviation $\hat{\sigma}$ and the standard deviation of the individual effect sizes for each psycholinguistic predictor m in Table D3.

D.2 Population-level effects of psycholinguistic predictors in the cross-method model

Figure D1 presents the population-level posterior effect estimates for various psycholinguistic predictors in the cross-method models.

Similar to the global intercept terms in Section 4.1.1, the cross-method data also show positive coefficients between 5.3 and 5.7. Word length and lexical frequency both exhibit positive effects, indicating that longer words lead to increased reading times across methods. However, in the case of lexical frequency, the 95% CrIs are wide and include zero, suggesting that these effects

Measure	Sample Sizes			Parameters									
	n_{obs}	n_{ind}	$\hat{\sigma}$	γ_{wl} local	γ_{wl} spill	γ_f local	γ_f spill	γ_s local	γ_s spill	γ_d local	γ_d spill	γ_{nl} local	γ_{nl} spill
FFD	440060	131	.41	.04	.04	.03	.03	.01	.01	.01	.02	.02	.02
				.04	.04	.03	.03	.01	.01	.02	.02	.02	.02
FPRT	380626	131	.44	.11	.06	.05	.03	.02	.01	.02	.03	.03	.02
				.11	.05	.04	.03	.01	.01	.02	.03	.02	.02
RPD	379581	131	.55	.11	.04	.04	.03	.03	.01	.03	.02	.02	.01
				.10	.04	.04	.03	.02	.01	.03	.02	.02	.02
TFT	438880	131	.50	.11	.05	.04	.02	.03	.01	.03	.02	.02	.02
				.10	.05	.04	.03	.02	.01	.03	.02	.02	.02
SPR	285273	65	.35	.10	.08	.05	.04	.03	.03	.03	.04	.02	.01
				.07	.08	.03	.02	.02	.03	.02	.03	.01	.01

TABLE D3 Overview of residual standard deviation ($\hat{\sigma}$) and between-subjects standard deviation of individual effects—both local and spillover—of psycholinguistic predictors $\hat{\sigma}_m$ for each session. $\hat{\gamma}_m$ represents the ratio $\frac{\hat{\sigma}_m}{\hat{\sigma}}$. Due to space reasons, $\hat{\sigma}_m$ is not included in the table but can be directly recovered from $\hat{\gamma}_m$ and $\hat{\sigma}_m$. n_{obs} denotes the total number of data points used for model fitting, n_{ind} the number of individuals. We only report the values for models fitted on continuous response variables as the ones fitted on binary or count-based response variables do not include a dispersion parameter. For each reading measure, the first row denotes the first session and the second row the second session. For models fitted on cross-method data, the first row denotes statistics for the self-paced reading (SPR) sessions, the second row the statistics for the eye-tracking sessions. Note that for the SPR sessions, the metrics are based on the spillover effects (see Section 4.2).

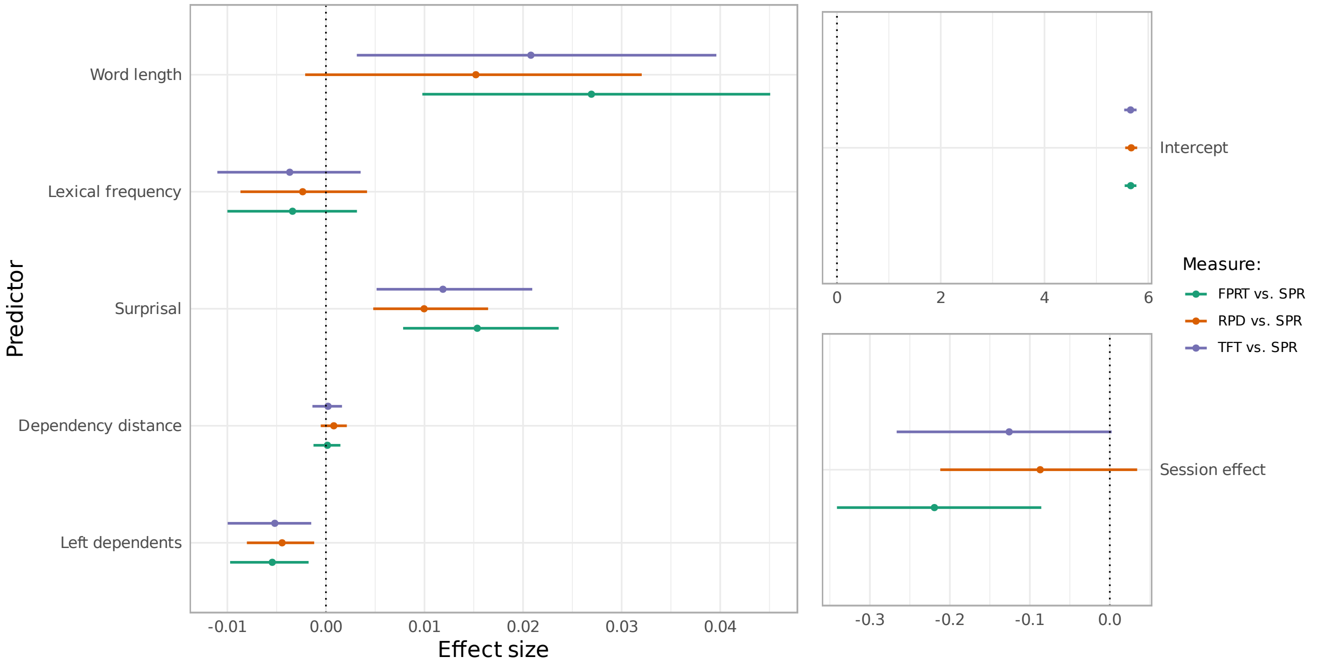

FIGURE D1 Population-level effects in cross-method models: population-level posterior distributions of the coefficients of different psycholinguistic predictors as well as the global intercepts and the session effect, observed with eye-tracking (FPRT, RPD, TFT) and self-paced reading. Effect sizes are in log space. We report each reading measure's mean estimate (represented by the dot) and 95% credible interval (the bar).

are statistically inconclusive at the population level. On the other hand, surprisal consistently increases reading times across all measures, with narrow credible intervals, providing stronger evidence for its effect.

Regarding syntactic integration, dependency distance clearly affects reading times in model fitted on RPD and SPR times, indicating that longer dependency distances lead to longer go-past times in eye-tracking and reaction times in self-paced reading. Although FPRT and TFT also show positive effects, the 95% CrIs include zero, indicating uncertainty in these estimates. The number of left dependents demonstrates clear negative effects across all three reading measures, suggesting that more left dependents reduce reading time on the head across methods.

D.3 Cross-method reliability: Comparison between local and spillover effects

In addition to the main analyses reported in Section 4.2, we directly compared cross-method reliability estimates obtained when comparing *local* effects in eye-tracking measures with *local* rather than *spillover* effects in self-paced reading (SPR). As shown in Figure D2, correlations between individual-level local effects in eye-tracking and local effects in SPR were generally low, suggesting poor reliability when strictly matching the temporal position of predictors across methods. By contrast, correlations were considerably higher when comparing local effects in eye-tracking with spillover effects in SPR.

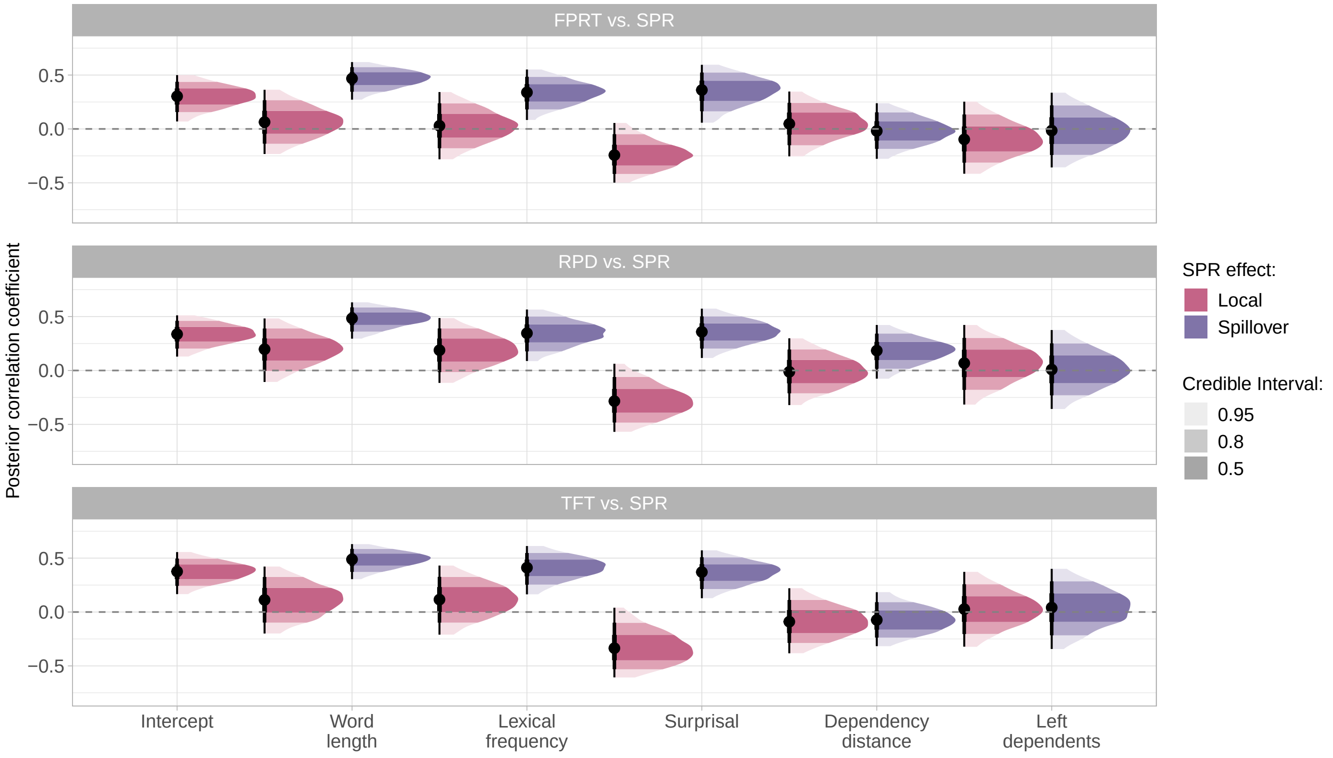

FIGURE D2 Comparison between correlation coefficients for cross-method reliability using local versus spillover effects: Posterior distributions of the Pearson correlation coefficients for cross-method reliability of the different psycholinguistic predictors and the intercept. For each of these predictor variables, correlations are computed between the by-subject random effects obtained from one of the eye-tracking measures (first-pass reading time FPRT, regression-path duration RPD, total fixation time TFT) and the by-subject random effects obtained from the SPR reaction times. For all coefficients except the intercept, we show both the correlation between the effect in response to the current word's predictor variable in eye-tracking and the effect in response to the current word's predictor variable in self-paced reading ("local") as well as the correlation between the effect in response to the current word's predictor variable in ET and the correlation between the effect in response to the *previous* word's predictor variable in self-paced reading ("spillover"). For each posterior distribution, the 50-, 80- and 95% credible intervals are shown.

References

Hagmann-von Arx, P., & Grob, A. (2014). *Reynolds Intellectual Assessment Scales and Screening: Deutschsprachige Adaptation der Reynolds Intellectual Assessment Scales (RIAS) & des Reynolds Intellectual Screening Test (RIST) von Cecil R. Reynolds und Randy W. Kamphaus*. Bern, Switzerland: Hogrefe.

Krakowczyk, D. G. (2023). *Python WMC Battery, version 1.1.0.* Zenodo. doi: 10.5281/zenodo.8310965

Lehrl, S. (2005). *Mehrfachwahl-Wortschatz-Intelligenztest MWT-B*. Balingen, Germany: Spitta Verlag.

Lewandowsky, S., Oberauer, K., Yang, L.-X., & Ecker, U. K. (2010). A working memory test battery for MATLAB. *Behavior Research Methods*, *42*(2), 571–585.

Moll, K., & Landerl, K. (2010). *SLRT-II: Lese-und Rechtschreibtest; Weiterentwicklung des Salzburger Lese- und Rechtschreibtests (SLRT).* Bern, Switzerland: Huber.

Moosbrugger, H., Oehlschlägel, J., & Steinwascher, M. (2011). *Frankfurter Aufmerksamkeits-Inventar 2: FAIR-2* (Second ed.). Bern, Switzerland: Huber.

Rouder, J. N., & Mehrvarz, M. (2024). Hierarchical-model insights for planning and interpreting individual-difference studies of cognitive abilities. *Current Directions in Psychological Science*, *33*(2), 128–135.

Stroop, J. R. (1992). Studies of interference in serial verbal reactions. *Journal of Experimental Psychology: General*, *121*(1), 15–23.
